# Recycling of End-of-Life Crystalline Silicon Photovoltaic Modules: A Comprehensive Review of Technologies, Challenges, and Prospects

**DOI:** 10.3390/molecules31162933

**Published:** 2026-08-21

**Authors:** Huide Fu, Yang Zhou, Bing Bai

**Affiliations:** 1 College of Chemistry and Environmental Engineering, Shenzhen University, Shenzhen 518060, China; 2 CGN Environmental Protection Industry Co., Ltd., Shenzhen 518000, China

**Keywords:** photovoltaic, end-of-life, recycling technologies, delamination

## Abstract

As global photovoltaic (PV) installation capacity grows rapidly, the environmental pollution and resource waste from the large-scale end-of-life (EOL) wave have drawn increasing attention. Traditional disposal methods such as landfilling and incineration are no longer viable, making green recycling a logical path for the PV industry. This paper reviews recent progress in the disassembly and recycling of EOL crystalline silicon (c-Si) PV modules. It first describes the structural material composition of c-Si PV modules and summarizes global recycling policies and regulatory frameworks. It then analyzes the mechanisms and process parameters of major delamination technologies, including mechanical crushing, pyrolysis, thermal cutting, high-voltage pulse fragmentation, solvent-based approaches, and laser peeling. Methods for purifying silicon and recovering precious metals such as silver and copper are also covered. Finally, key challenges in the recycling field and future development trends are discussed, with the aim of supporting the advancement of c-Si PV recycling technologies and the sustainable development of related industrial chains.

## 1. Introduction

**Figure 1:**
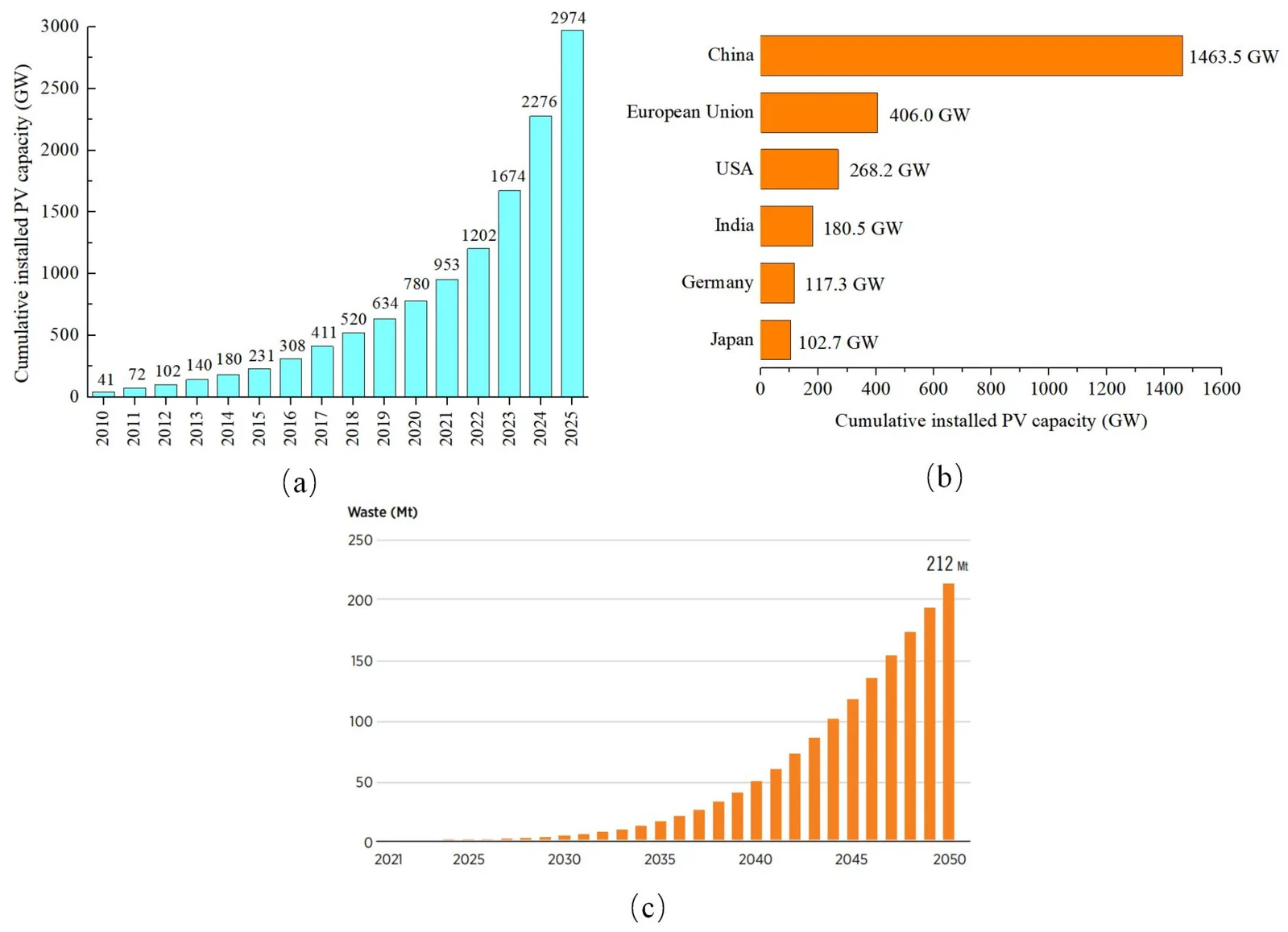
(**a**) The global cumulative PV installed capacity of EOL PV modules and (**b**) Top 6 cumulative installed capacity in 2025. Data sourced from De l’Epine et al. [1] (IEA PVPS Task 1, T1-49, April 2026); (**c**) Global cumulative EOL PV module waste from 2021 to 2050. Reproduced from IRENA [2] (World Energy Transitions Outlook 2022: 1.5 °C Pathway, Abu Dhabi: IRENA, 2022 [2]) under CC BY-SA 3.0 IGO; permission via IRENA Publications.

The global energy structure is accelerating its transition towards low-carbon development. As one of the most promising renewable energy technologies, solar PV power generation continues to exhibit exponential growth in installed capacity. According to statistics from the International Energy Agency (IEA), the global cumulative installed PV capacity surged from 41 GW in 2010 to 2974 GW in 2025 over the past fifteen years (Figure 1a) [1], with c-Si PV modules accounting for the vast majority of this total. This rapid expansion underscores the critical role of PV technology in addressing global climate change, promoting the decarbonization of the energy mix, and reducing greenhouse gas emissions. In terms of geographical distribution, PV installed capacity is highly concentrated in a few major markets, such as China, the European Union, the United States, India, Germany, and Japan. In particular, by the end of 2025, China’s cumulative installed PV capacity reached approximately 1463.5 GW, accounting for nearly 50% of the global cumulative total (Figure 1b) [2].

PV modules typically have a service life of 25–30 years. As early installations progressively reach their EOL, the world is facing a massive wave of PV module retirements. In addition, some modules may be decommissioned earlier due to inadequate maintenance, early-stage quality issues, extreme weather events, damage during transportation and installation, or technological upgrades [3,4]. According to forecasts by the China Photovoltaic Industry Association, China will begin to see large-scale retirement of PV modules from 2025. According to the International Renewable Energy Agency (IRENA) projections, waste from cumulative global solar PV projects is expected to grow from 0.2 million tonnes (Mt) in 2021 to 4 Mt by 2030, then to nearly 50 Mt by 2040, and ultimately exceed 200 Mt by 2050 (Figure 1c) [2]. G20 member countries are expected to account for nearly 90% of this waste [2]. Confronted with such a huge quantity of EOL PV modules, conventional disposal methods such as landfilling or incineration are no longer viable. Discarded PV modules contain toxic substances such as lead, cadmium, and fluorides, which, if not properly handled, can migrate through soil and groundwater, causing persistent ecological pollution [5,6,7]. At the same time, these modules are rich in recoverable materials such as glass, aluminum, silver, copper, and silicon. Efficient recycling could therefore generate substantial economic value. Research by Deng et al. shows that using silicon wafers recycled from decommissioned PV modules to manufacture new solar cells can save approximately 20% in manufacturing costs [8]. Additionally, for every 100 kg of decommissioned PV modules, metals worth about $72 can be recovered [9]. Therefore, promoting environmentally sound dismantling and resource recovery of PV modules supports pollution prevention and resource circulation, and directly contributes to the sustainable development of the PV industry and the broader transition toward a green, low-carbon economy. The case for recycling is therefore clear.

This review introduces the structure and material composition of the c-Si PV modules, reviews and analyzes existing treatment methods for EOL c-Si PV panels, and underscores the necessity for large-scale recycling. Building on this foundation, the study systematically summarizes the main technological pathways for dismantling and resource recovery from decommissioned c-Si PV modules, and outlines the key technical, economic, and environmental challenges confronting the practical implementation of current recycling technologies. Furthermore, the future development trends in the field are also discussed, with the aim of providing theoretical references and practical insights to support the advancement of PV recycling technologies and the sustainable development of related industrial chains.

## 2. Types and Composition of c-Si PV Modules

### 2.1. Types of c-Si PV Modules

**Figure 2:**
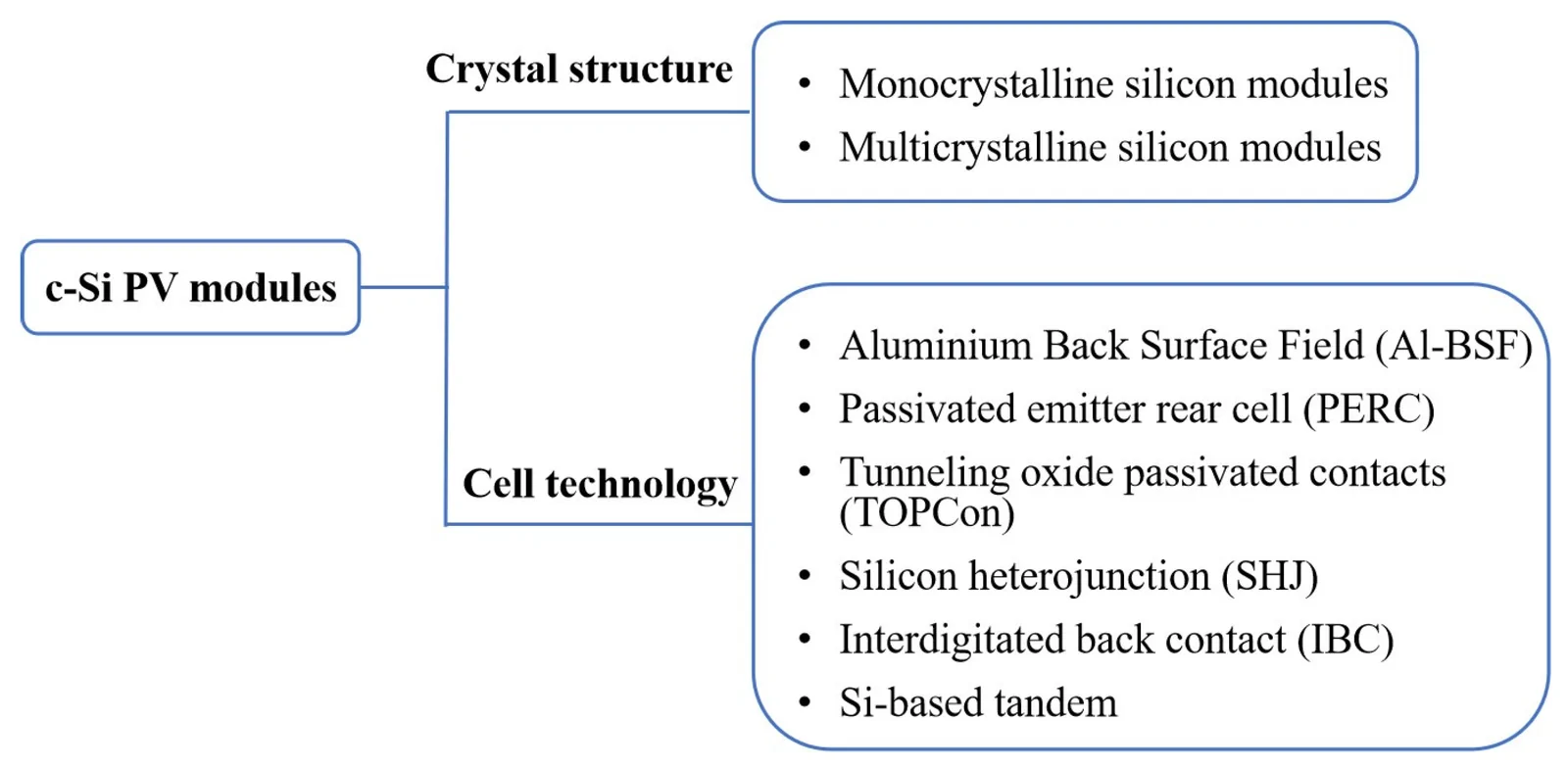
Classification of c-Si PV modules.

As shown in Figure 2, based on the crystal structure of the silicon wafers, c-Si PV modules can be classified into monocrystalline silicon (mono-Si) and multicrystalline silicon (mc-Si) modules. Additionally, c-Si PV modules also can be further subdivided into six subtypes based on different cell technologies [10]: (1) aluminum back surface field (Al-BSF); (2) passivated emitter and rear cell (PERC); (3) tunnel oxide passivating contact/polysilicon on oxide (TOPCon/POLO), among which TOPCon is the most commonly used name for this technology; (4) silicon heterojunction (SHJ); (5) interdigitated back contact (IBC), which includes metal-wrap-through designs; and (6) Si-based tandem.

Currently, PV module products based on mc-Si have completely disappeared from the market due to their lack of competitiveness. Meanwhile, with the continuous advancement of cell technologies, early PV modules based on Al-BSF technology also exited the market several years ago. During the period from 2018 to 2023, PV modules based on PERC technology experienced a significant increase in market share and once became the mainstream technology [10]. However, over time, the mass-production efficiency of PERC has gradually approached its theoretical limit, leaving almost no room for further improvement. Against this backdrop, PV modules based on n-type TOPCon technology have demonstrated significant structural advantages. Its passivating contact structure effectively reduces rear-side recombination losses, opening up new headroom for efficiency enhancement [10,11]. At the same time, TOPCon exhibits high compatibility with PERC production lines, resulting in relatively low retrofitting costs. These factors have enabled TOPCon to rapidly replace PERC technology. Currently, TOPCon has become the dominant technology in the photovoltaic market and is expected to maintain a high market share in the coming years. In addition, emerging technologies such as SHJ, IBC, and Si-based tandem solar cells are also advancing in parallel and are expected to further drive technological iteration and efficiency breakthroughs in the photovoltaic industry in the future [11].

### 2.2. Structures of c-Si PV Modules

**Figure 3:**
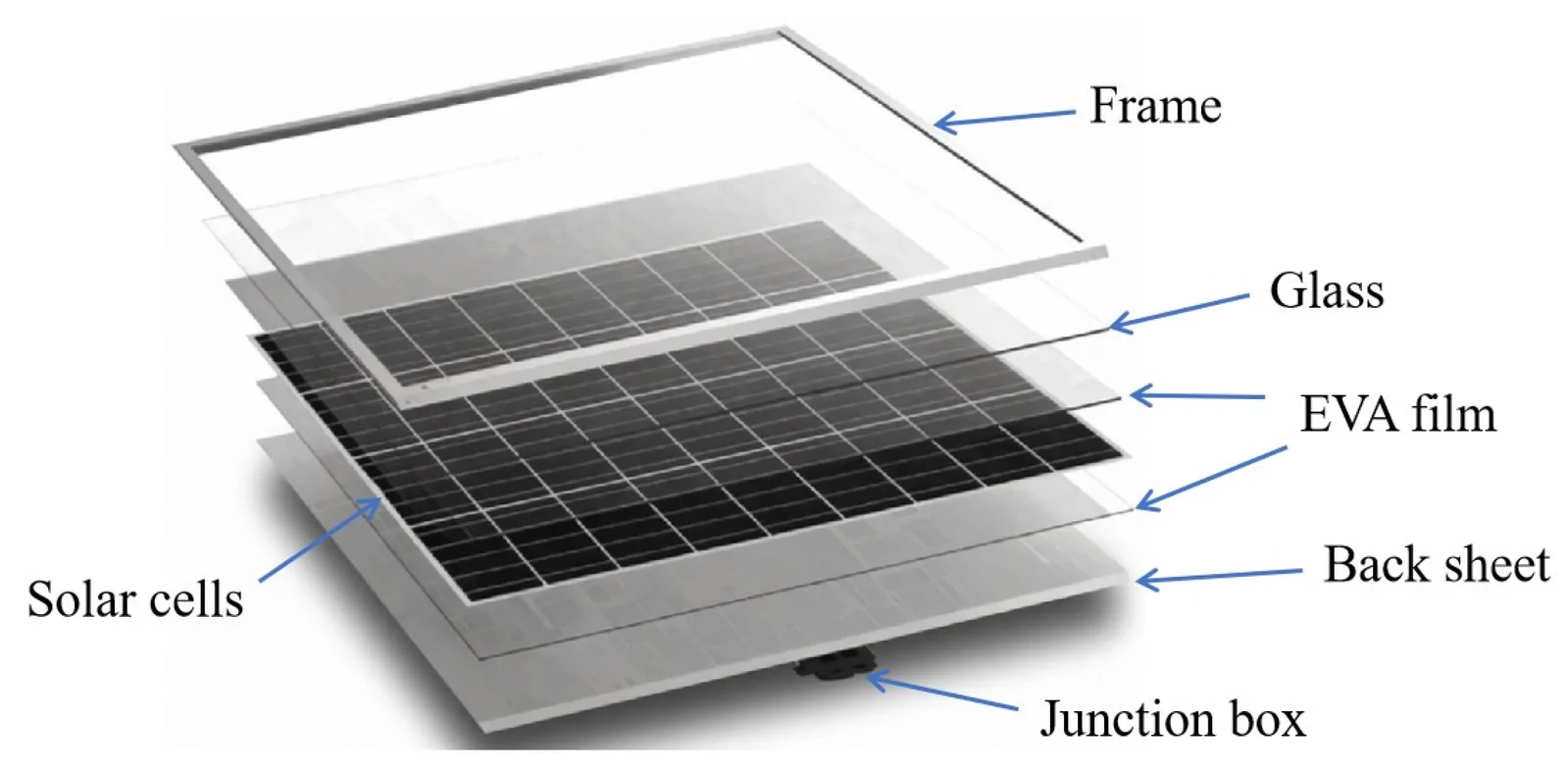
Structure of typical c-Si PV modules.

The structure of a typical c-Si PV module is shown in Figure 3. The c-Si PV modules feature a multi-layered, sandwich-like architecture. The outermost component is a robust Al alloy frame, which provides essential structural integrity and protection. When viewed from top to bottom, the top layer consists of tempered glass, serving as a highly transparent and durable front shield. Immediately beneath the glass lies a layer of polymer encapsulant, typically ethylene vinyl acetate (EVA). This material acts as a critical adhesive and barrier, bonding the subsequent layers together to prevent damage from thermal, mechanical, ultraviolet, and moisture exposure [6]. The core functional unit, the crystalline silicon photovoltaic cell with a thickness of 130–150 µm, is situated in the middle of the PV module, and the photoelectric conversion process occurs here. The PV cell is fabricated from a silicon wafer doped to form an n-p junction. Its top surface is coated with an anti-reflective (AR) layer (composed of materials like SiO and SiO_2_ or Si_3_N_4_ and Al_2_O_3_ [12,13]) to maximize light absorption. Electrical contacts are formed by screen-printed or laser-grooved silver gridlines on the top side and aluminum or silver electrodes on the bottom side. Individual cells are interconnected using solder ribbons, which feature a copper core with tin-lead plating. Beneath the solar cell array lies another layer of polymer encapsulant, which serves to bond the solar cells to the backsheet. The rearmost layer is the backsheet, often a laminate incorporating fluorinated polymers such as Tedlar^®^ (PVF) or PVDF, which serves to protect the internal components, such as solar cells, from environmental erosion (e.g., heat and humidity) and to provide electrical insulation. The entire assembly is functionally integrated with a junction box, typically attached to the backsheet, which serves as the central node for converting, storing, and transmitting the generated electrical energy.

Additionally, it should be noted that, with the continuous improvement of technology, the structure of c-Si PV modules will undergo some changes, mainly reflected in the following aspects. First, bifacial modules are adopting glass backsheets to replace traditional PVDF backsheets, thereby enhancing light transmittance and bifacial power generation gain. Second, in terms of encapsulation materials, Polyolefins (POE) or EVA/POE co-extruded films are gradually replacing single-layer EVA films to improve anti-Potential Induced Degradation (PID) performance and weatherability. Third, the size of solar cells continues to increase, while the thickness of silicon wafers is further reduced, aiming to achieve higher module power output and lower material costs [11].

### 2.3. Material Components of c-Si PV Modules

The weight percentage of various material components in c-Si PV modules varies across literature sources [14,15,16]. Moreover, the composition of these materials continuously evolves with advancements in PV technology. Table 1 presents the material composition of c-Si PV modules. The data show that glass is the dominant component, with its mass fraction ranging from 69% to 76%. In addition, c-Si PV modules contain various metals, including the precious metal silver as well as base metals such as copper, aluminum, tin, and lead. Although silver accounts for a very small mass fraction in the module, its economic value is notable. Furthermore, materials such as copper, aluminum, and silicon also exhibit significant recycling potential. Despite the low unit price of glass, its large mass share in the module ensures that it retains economic value during the recycling process of EOL c-Si PV modules. Lead and certain organic polymer materials require controlled disposal to avoid harming the environment and human health.

## 3. Management and Treatment of EOL c-Si PV Modules

### 3.1. Treatment of EOL c-Si PV Modules

**Figure 4:**
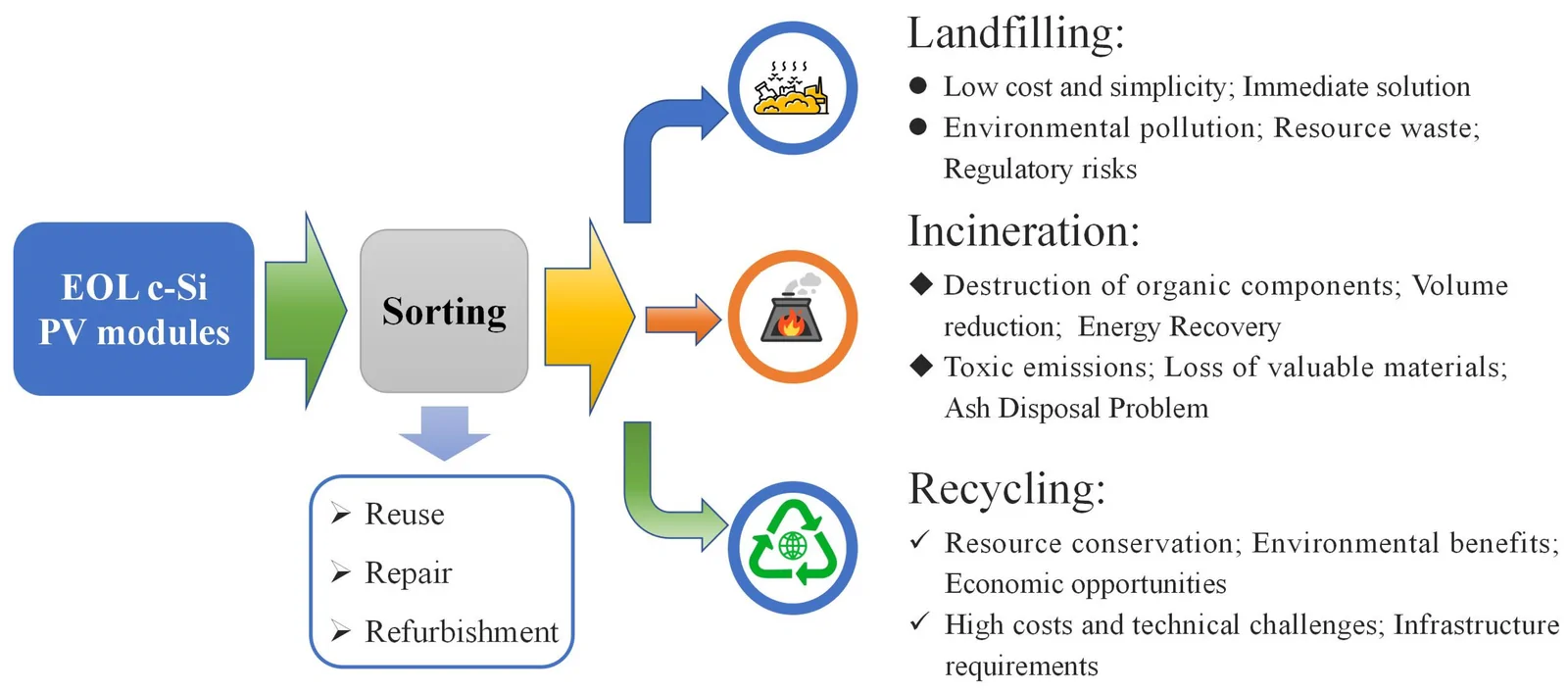
Treatment process of EOL c-Si PV modules.

Currently, the overall treatment pathways for EOL c-Si PV modules are illustrated in Figure 4. First, EOL c-Si PV modules are sorted to identify those that can be directly reused or reused after simple repair, allowing them to enter the circular reuse stage. For example, in Japan, the total amount of PV module waste collected in 2022 was 2079 tonnes, of which 441 tonnes were reused [17]. For modules that cannot meet the requirements for reuse, repair, or refurbishment, they are directed to landfill, incineration, or recycling treatment pathways, depending on their condition and local regulations. However, from a global perspective, EOL c-Si PV modules in many countries and regions are still primarily managed through landfill or incineration [18,19,20]. This is largely because the current volume of retired PV modules generated in these countries and regions has not yet reached a scale sufficient to form a substantial waste stream, and thus has not attracted sufficient attention. Furthermore, from an economic feasibility standpoint, establishing sustainable PV material recycling systems in most regions still faces challenges related to cost-effectiveness [21].

According to the IEA Photovoltaic Power Systems Programme (IEA-PVPS) report [22], the routing of EOL PV modules into direct reuse, repair/refurbishment, second-life application, or material recycling is determined by a four-step triage:

① Desktop Eligibility Screening:

Based on historical monitoring data, two criteria—performance loss rate (PLR) and residual value—are used to preliminarily screen batches of modules that require further inspection.

② On-Site Inspection and Functionality Testing:

Visual inspection is performed to exclude modules with obvious physical damage such as broken glass, cracked backsheets, or detached junction boxes. I-V curve tracing and infrared imagery are used to measure actual maximum power output and detect internal defects (e.g., microcracks, hot spots), respectively. Testing standards follow IEC 62446-1 [23] and IEC TS 62446-3 [24].

③ Sampling Lab Testing:

Laboratory tests may include I-V characterization (flash) tests, lock-in thermography (LIT), electroluminescence (EL) imaging, diode tests, and climatic chamber aging tests, etc.

④ Safety Testing:

Ground continuity (MST 13) and insulation resistance (MST 16) tests are conducted in accordance with IEC 61730-2 [25], supplemented by wet leakage current testing (MST 17) when necessary.

After the above testing and evaluation, the PV modules are classified according to the following criteria:

Direct Reuse: Modules with no apparent defects or only minor cosmetic imperfections (e.g., slight scratches, encapsulant or backsheet discoloration) that do not affect functionality, and that pass all safety tests.

Repair/Refurbishment Followed by Reuse: Modules with repairable defects, including failed bypass diodes, damaged junction boxes or cabling, localized backsheet microcracks, or power loss below 10% caused by snail trails or cracked cells. After repair and re-testing, these modules may enter the second-life market.

Material Recycling: Modules with irreparable damage, including broken glass, hot spots/burn marks, severe backsheet delamination, broken interconnects or poor soldering, corrosion, cracked cells causing power loss exceeding 10%, or modules that fail safety tests due to serious electrical hazards.

No unified international certification standard currently exists for second-life PV modules. In practice, requalification evidence is provided through traceability records and test documentation (e.g., I-V data, insulation results, EL images) issued by the assessing entity [22]. The IEC technical committee is developing a related Publicly Available Specification (PAS), which has not yet been released. On the economic side, new module prices keep falling while repair of decommissioned modules remains mostly labor-intensive and hard to automate. As a result, second-life applications are economically viable mainly in remote areas or under policy support. Overall, second-life application of decommissioned PV modules faces several challenges [22]: (1) high heterogeneity of module specifications, complicating system integration and grid compliance; (2) risk of secondary damage during transport and storage; (3) lack of large-scale automated testing facilities, limiting inspection throughput; (4) low economic viability under normal conditions; and (5) absence of standardized pass/fail criteria.

For EOL c-Si PV modules that cannot meet the requirements for second-life application, although landfilling and incineration are currently the lowest-cost treatment options, these approaches readily lead to the release of harmful substances into the environment, causing pollution and posing potential negative impacts on ecosystems and human health. This is because PV modules typically contain hazardous components such as lead, cadmium, and other heavy metals. If these substances leach into soil and water systems, they can pose serious threats [26,27] and, through bioaccumulation and biomagnification, present potential risks to plants, animals, and human health [28,29], while also incurring external environmental costs related to air, water, and soil pollution [20,30]. Furthermore, the high-temperature environment during incineration can promote the release of toxic gases like dioxins and furans, exacerbating air pollution and causing harm to human health [31]. Research by Uctug et al. [32] indicates that landfilling silicon and wafer waste generated during PV manufacturing can contribute up to 95% to the human toxicity potential. Beyond environmental risks, landfilling results in the loss of substantial reusable resources, including glass, aluminum, and rare metals such as silver, leading to significant economic losses [33,34]. A life-cycle assessment study by Faircloth et al. [26] revealed that the largest environmental impact from landfilling is observed in the metal depletion category, as valuable materials like copper, silver, aluminum, and silicon are lost in the process. This forces a return to the original, energy-intensive, and environmentally burdensome production processes to replace these materials, further increasing the environmental load. Given both environmental and resource considerations, landfilling and incineration are therefore not suitable treatment methods for EOL PV modules.

### 3.2. Regulations and Policies for EOL Management of PV Modules

Recycling EOL PV modules reduces environmental pollution, conserves land needed for landfilling, and enables circular utilization of valuable materials such as aluminum, silver, silicon, and glass, yielding both environmental and resource benefits. Currently, major global PV manufacturing and utilizing countries have successively introduced relevant laws and regulations, technical standards, or management frameworks to promote the regulated disposal and resource recovery of decommissioned PV modules.

#### 3.2.1. Europe

In 2012, the EU adopted the “Waste Electrical and Electronic Equipment” (WEEE) Directive (2012/19/EU), mandating the recycling of PV modules [35]. This directive establishes binding targets for the collection, recycling, and recovery of WEEE. By 2012, all EU Member States had implemented the PV regulation into national law, requiring PV manufacturers to either operate their own take-back systems or join producer compliance schemes. Under the principle of Extended Producer Responsibility (EPR), producers or their authorized representatives shall recover WEEE using best available techniques, and shall also finance the collection, treatment, recovery, and environmentally sound disposal of WEEE originating from private households. The WEEE Directive from 2019 sets minimum annual collection rates at 65% of the average weight of EEE placed on the market over the past three years, or 85% of WEEE generated domestically. Furthermore, it mandates a minimum 85% recovery rate and an 80% recycling or reuse/preparation for reuse rate. Notably, the European Committee is currently developing a “Recyclability Index” of PV modules as a prospective criterion for future Ecodesign standards [20].

Under the unified framework of the aforementioned WEEE Directive, European countries have enacted domestic decrees with varying emphases regarding the recycling management of photovoltaic modules. The following section provides an overview of the relevant regulations and mechanisms in some European countries.

① Germany

Germany revised its “Electrical and Electronic Equipment Act” (ElektroG) in October 2015, adopting the recovery and recycling rate requirements of the EU WEEE Directive [36]. Under this Act, PV module manufacturers bear full responsibility for the products they place on the market, covering take-back, logistics, sorting, dismantling, recovery, and recycling, and must provide corresponding financial guarantees. The German government has established different collection and take-back regulations based on the type of PV module application—business-to-business (B2B) or business-to- consumer (B2C)—to manage electronic waste from PV panels accordingly. Under the B2C mechanism, current collection and recycling costs (including historical decommissioned modules) are shared among existing manufacturers in proportion to their current market share. Financial security for future waste collection and recycling is guaranteed by manufacturers through declarations made at the time of registration; if a manufacturer exits the market, other manufacturers must assume its corresponding share of responsibility. Under the B2B mechanism, manufacturers or their authorized representatives must bear the take-back and recycling costs for modules placed on the market after 24 October 2015, while for historical modules, the end users bear the corresponding costs.

② France

France transposed the EU WEEE Directive (2012/19/EU) into its national law through Decree No. 2014-928 of 2014, incorporated into the “French Environmental Code” [37]. France implements an EPR system, administered by Producer Responsibility Organizations (PROs). Soren is a non-profit PRO approved by the French Ministry of the Environment, responsible for the centralized collection and recycling management of photovoltaic waste [38], and authorizes treatment operators to carry out specific tasks through a tendering process. According to the 2020 Anti-Waste Law and the revised EPR regulations of 2021 [39], France has established the following quantitative targets: a collection target of 65% of the average weight of equipment placed on the market over the previous three years, or 85% of WEEE generated in the country; a minimum recovery rate of 87% and a minimum recycling rate of 82% since 2024; and a reuse target of 2% since 2023.

③ Italy

Italy transposed the EU WEEE Directive (2012/19/EU) into its national regulatory framework through Legislative Decree No. 49/2014 [40]. The decree requires producers to achieve a minimum recycling rate of 85% for photovoltaic modules, and classifies end-of-life PV modules into household and professional categories based on installed capacity, with differentiated management applied accordingly. Furthermore, pursuant to Article 1 of Legislative Decree No. 118/2020 and the amendments introduced by Laws No. 41/2023 and No. 87/2023, Italy’s energy services manager issued new guidelines for the disposal of modules installed in plants benefiting from feed-in tariff (FIT) schemes [20]. For domestic modules that have reached end-of-life, they can be processed by delivery to national collection centers or through the distributor/installer from whom the modules were originally purchased. For professional PV modules, once decommissioned, they can be handed over to authorized waste treatment entities, transported to authorized treatment facilities, or delivered to the national collective recycling system.

④ Spain

Spain formally transposed the EU WEEE Directive (2012/19/EU) into national law through Royal Decree 110/2015 of 20 February 2015 [41]. This decree requires producers (including manufacturers, distributors, installers, etc.) to fulfill the management obligations for their product waste and bear the corresponding management costs. Producers must register their products in the “National Register of Producers of Electrical and Electronic Equipment”. Subsequently, pursuant to the amendments to Royal Decree 208/2022, Spain classified PV modules into three categories: Silicon non-hazardous, other non-hazardous, and hazardous. In January 2023, the Spanish Waste Coordination Commission further specified that the treatment of EOL PV modules should not be limited exclusively to thermal treatments or equivalent techniques, but may also adopt mechanical or chemical treatment methods [42].

#### 3.2.2. Japan

Japan manages EOL PV modules under the general industrial waste framework of the “Waste Management and Public Cleansing Act”. This law defines the scope of waste and clarifies the responsibilities of industrial waste generators and treatment operators, covering various industrial waste treatment requirements including landfill disposal. Building on this foundation, in August 2024, the Ministry of Environment (MOE) published the third edition of the “Guidelines for Promoting Proper End-of-Life Treatment (Including Recycling) of Photovoltaic Modules” [43]. These guidelines provide basic information on relevant laws and regulations concerning decommissioning, transportation, reuse, recycling, and industrial waste disposal. To further advance the establishment of a mandatory recycling system for EOL PV modules, a working group jointly organized by the Ministry of Economy, Trade and Industry (METI) and the MOE drafted a regulatory concept in March 2025. This concept proposes a mandatory recycling mechanism covering materials, costs, and information, along with a producer-paid scheme managed by an independent third-party organization [44].

#### 3.2.3. South Korea

The Ministry of Environment of South Korea promulgated the “Electrical and Electronic Equipment and Vehicle Resource Recycling Act” in 2019, which includes PV modules among 23 product categories subject to extended producer responsibility, with related obligations taking effect from 2023 [45]. The Korean non-profit organization E-Cycle Governance, as the exclusive operator for PV module waste EPR, has been responsible for the overall collection and recycling of PV module waste since January 2023 [20]. Companies performing the actual collection and recycling must register as members of E-Cycle Governance and receive subsidies based on their processing performance. In the same year, the Korean government confirmed the “Plan to Strengthen Management of PV waste Panels”, jointly formulated by the Ministry of Environment and the Ministry of Trade, Industry and Energy. The plan covers six key areas: manufacturing (promoting resource-circulating), dismantling (strengthening safety management), collection (establishment of collection systems based on waste volume and disaster situations), processing (establishing regional collection and recycling systems), waste reduction (establishing a basis for reuse of usable modules), and infrastructure (improvement of statistics and information provision). It aims to promote full-chain resource circulation from production to decommissioning, prevent the abandonment of waste modules, reduce logistics and treatment costs, and ultimately achieve a recycling/reuse rate of over 80% within three years [20].

#### 3.2.4. China

At the regulatory level in China, the Law of the “People’s Republic of China on the Prevention and Control of Environmental Pollution by Solid Waste” serves as the primary legal basis for classifying and managing hazardous substances and solid waste from retired PV modules. Additionally, in 2023, the National Development and Reform Commission, together with five other government agencies, jointly issued the “Guiding Opinions on Promoting the Circular Utilization of Retired Wind Power and Photovoltaic Equipment”, which clarifies that centralized PV power station operators bear the statutory responsibility for the circular utilization and proper disposal of retired modules, while encouraging manufacturers to establish distributed recycling systems and provide third-party “one-stop” services [46]. Regarding the specific treatment of retired PV modules, in accordance with the “General Specification for Echelon Use of Decommissioning Photovoltaic Modules” (GB/T 45075-2024) [47], which took effect in June 2025, priority is given to the cascaded utilization of retired modules. Modules that are not suitable for cascaded utilization proceed to the dismantling and regeneration stage. Meanwhile, the “Technical Specification for Pollution Control of Waste Photovoltaic Equipment Recycling and Treatment” (HJ 1463-2026) [48], issued by the Ministry of Ecology and Environment in January 2026, systematically regulates pollution prevention and control throughout the entire process of dismantling, collection, transportation, storage, disassembly, comprehensive utilization, and disposal.

#### 3.2.5. United States of America

In the United States, there are no PV waste-specific regulations; EOL PV modules are managed under the “Resource Conservation and Recovery Act” (RCRA) [21]. In October 2023, the U.S. Environmental Protection Agency initiated a rulemaking process to classify PV modules as universal waste [49], recognizing that they pose less harm to human health and the environment compared to other hazardous wastes, thereby allowing for less stringent collection restrictions and management requirements. Currently, three U.S. states have enacted specialized regulations governing the EOL management of PV modules. California (SB 489) [50] and Hawaii (HAR 11-273.1) [51], effective in 2021 and 2025, respectively, have designated PV modules as “universal waste” to promote the regulated management of modules containing hazardous materials; such modules may be recycled or landfilled at designated universal waste treatment facilities. In 2017, Washington State passed ESSB 5939, requiring PV manufacturers to provide free take-back services for EOL modules and achieve an 85% weight-based reuse or recycling rate. However, due to implementation challenges and industry resistance, the state enacted SB 5175 in April 2025 to defer enforcement: manufacturers must submit product stewardship (or producer responsibility) plans by 2030, and starting in 2031, only PV modules covered by an approved plan (including free collection and reuse/recycling targets) may be sold [52].

#### 3.2.6. India

Currently, India lacks specific laws and regulations dedicated to the management of PV module waste, and such waste is handled under general waste management regulations [53]. However, in 2020, the Ministry of New and Renewable Energy (MNRE), in collaboration with the International Finance Corporation (IFC), developed the “National Programme on Solar PV Waste Management” [54]. This programme provides a framework for the sustainable management of EOL PV waste, covering aspects such as establishing a regulatory framework, capacity building, and public awareness raising, while also promoting a circular economy for PV waste, including the reuse, recycling, and extraction of valuable materials [55]. Additionally, MNRE has proposed the establishment of a corpus fund mechanism for the safe disposal of EOL PV waste. At the regulatory level, the Central Pollution Control Board (CPCB) is the authority responsible for overseeing the management of EOL PV waste in India. Solar PV manufacturers are required to submit an EOL management plan to the CPCB and obtain authorization; this plan must detail how PV waste modules will be collected, transported, stored, and disposed of, ensuring that waste modules are managed in an environmentally sustainable manner [55].

## 4. Recycling of c-Si PV Modules

**Figure 5:**
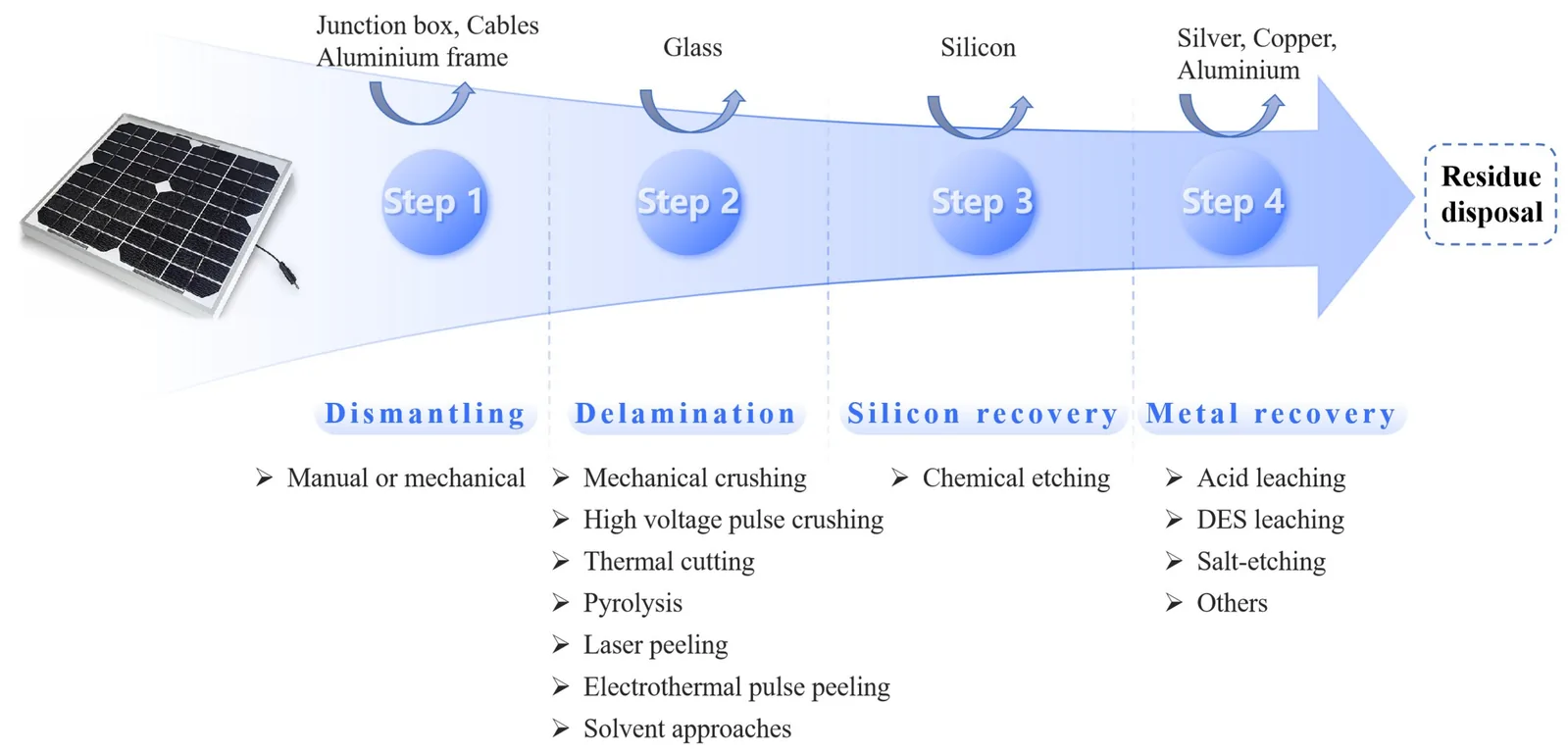
Recycling process of c-Si PV modules.

As shown in Figure 5, a complete c-Si PV module recycling pathway typically follows a process of disassembly, layer separation, and material extraction. Firstly, external components such as junction boxes, aluminum frames, and cables are removed manually or mechanically. Subsequently, the core laminate is delaminated to separate the glass, PV cells, backsheet, and encapsulant (e.g., EVA). This step is technically critical, requiring a balance between separation efficiency and material integrity. Finally, silicon and valuable metals such as silver, copper, and aluminum are purified and recovered.

Materials recovered from decommissioned PV modules can be directed to different reuse routes depending on the material type. Copper components from junction box terminals, cables, and ribbons can be remelted and reused as conductors for wires and cables; aluminum frames can be regenerated into ingots for manufacturing new PV frames, architectural aluminum profiles, or other structural aluminum components; and silver, as a critical precious metal in grid lines, can return directly to the silver paste supply chain after separation and purification. Regarding non-metallic and semiconductor materials, glass constitutes the largest mass fraction of a module (approximately 70%), and its utilization pathway depends on its integrity: intact glass sheets can be used in agricultural greenhouses and potentially the manufacture of new PV modules [20], while glass particles serve as downgraded raw materials for flat glass, foam glass, glass fiber, or concrete aggregate [20,56]. Furthermore, silicon materials, once chemically purified to high-purity silicon, hold the potential to be reused as raw materials for PV cells [57,58] or as anode materials for lithium-ion batteries [59,60], thereby achieving high-value recycling.

### 4.1. Methods of c-Si PV Module Delamination

Crystalline silicon PV modules require excellent sealing integrity and durability throughout their service life. To achieve this, processes such as high-temperature vacuum lamination are typically employed to form a robust composite structure between their layers, ensuring long-term stable performance [4]. The bonding strength usually in the range 30–40 N/cm for EVA–glass and >20 N/cm for EVA–backsheet [61,62]. This high-strength bonding effectively enhances the modules’ mechanical strength, waterproofing, and environmental adaptability. However, it also makes the disassembly process significantly more complex during EOL recycling.

Currently, the principal delamination methods for decommissioned c-Si PV modules include mechanical crushing, high-voltage pulse fragmentation, pyrolysis, thermal cutting, laser peeling, electrothermal pulse peeling, and solvent approaches. In addition, the combined or hybrid use of multiple methods has also developed into an important technical approach.

**Figure 6:**
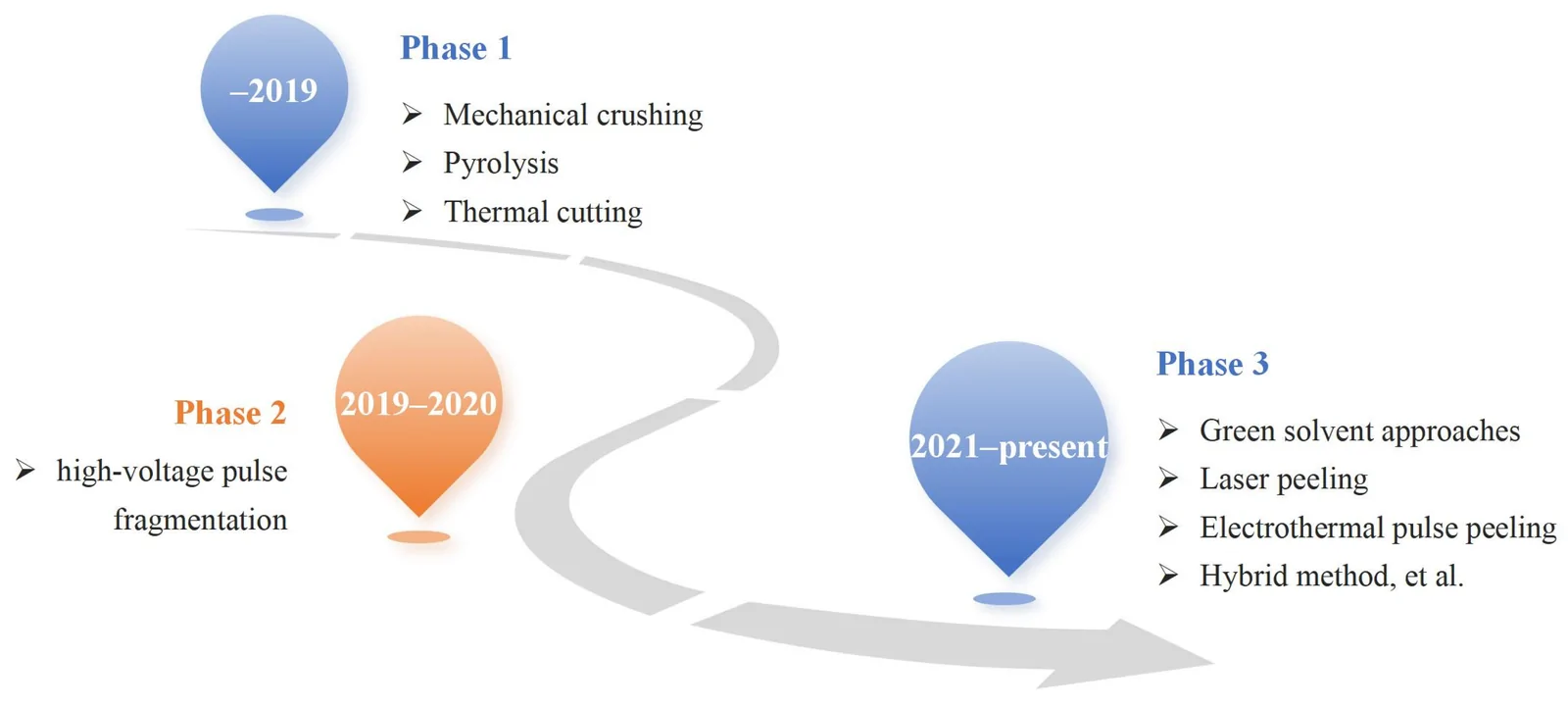
Evolution of delamination techniques in EOL c-Si PV module recycling.

As illustrated in Figure 6, a statistical review of the relevant research literature conducted by the authors reveals that the evolution of delamination technologies for decommissioned c-Si PV modules can be broadly categorized into three phases:

- Phase 1 (up to 2019): This phase was predominantly characterized by conventional mechanical and thermal treatment methods, such as mechanical crushing, pyrolysis, and thermal cutting, representing a relatively traditional technological approach.
- Phase 2 (2019–2020): During this period, the focus shifted toward physical pulse-based separation techniques, notably high-voltage pulse fragmentation, reflecting an increasing emphasis on material damage mitigation and selective fragmentation.
- Phase 3 (2021–present): This phase is marked by technological refinement and a pronounced shift toward green and environmentally sustainable approaches. Environmentally benign chemical/solvent technologies, particularly green solvent approaches, have gained prominence as important research directions. At the same time, selective delamination techniques have advanced along two distinct pathways: (i) beam-based precision peeling, such as laser ablation of the encapsulant; and (ii) thermal-pulse interface separation, such as electrothermal pulse peeling, which uses differential thermal contraction for nondestructive layer separation. Hybrid processes that integrate multiple methods have also been developed. Overall, the field is advancing toward efficient, low-damage, and environmentally sustainable recycling solutions.

#### 4.1.1. Mechanical Crushing

Mechanical crushing is currently one of the most mature methods for treating decommissioned c-Si PV modules. This process typically applies shear, impact, or compressive forces to the modules using equipment such as hammer mills, dual-blade rotor crushers, knife mills, or shear presses, followed by screening and classification (e.g., vibrating screens, heavy-medium separation, or air classification) to recover glass, silicon, and metals [56,63,64,65,66,67,68,69,70]. Table 2 summarizes the main process steps, operating parameters, recovered products, and key quantitative results reported in the literature.

The recovery performance of mechanical crushing varies with equipment configuration and the number of crushing stages. Hydraulic shearing combined with chain crushing, followed by particle-size-based screening and classification, can recover approximately 60% of metals from c-Si modules, yet the presence of glass increases sorting difficulty [63]. In contrast, combining crushing with grinding, together with heavy-medium separation and sieving, can concentrate 97% of glass into particles smaller than 5.6 mm [56], and achieve up to 76% glass recovery and nearly 100% metal recovery, with glass and metal grades of 100% and approximately 67%, respectively [64]. Multi-stage crushing (e.g., dual-blade rotor plus hammer mill) or repeated crushing (three or more cycles) enables effective separation of glass, polymers, and fine particles [65], improves glass recovery rates [66], and promotes selective enrichment of high-value silver in the finest particle fraction (accounting for over 70% of the total silver content) [67]. When further combined with electrostatic separation technology, metal components in the crushed waste can be efficiently enriched, achieving an enrichment rate of up to 95% [68]. In addition, mobile mechanical treatment systems, through on-site shearing, swing/fixed hammer milling, and multi-stage screening, can produce qualified glass cullet while concentrating silicon and metals, and reducing transportation costs [69]. However, purely mechanical routes generally struggle to effectively separate polymers and still require the integration of pyrolysis or solvent treatment to liberate encapsulated materials [65,66,70].

#### 4.1.2. Pyrolysis

Pyrolysis is a mature method for delaminating c-Si PV modules and removing polymer encapsulants such as EVA. By applying high temperatures to EOL PV modules after the removal of aluminum frames and junction boxes, EVA undergoes thermal decomposition or combustion, enabling the separation of glass, silicon, and metals. However, direct pyrolysis of intact modules generates hazardous fluorides and residues from the backsheet (e.g., Tedlar^®^) [71]; therefore, pre-removal of the backsheet is generally required. Existing backsheet removal methods include thermal processes (preheating at 90–200 °C using halogen lamps or hot plates to soften the EVA without causing decomposition) [72,73,74] and mechanical processes (grinding) [75]. Both pretreatment strategies can effectively shorten processing time, reduce harmful gas emissions, and minimize solid residues [72,73,74,75]. Table 3 summarizes the main process routes, parameters, and recovered products related to pyrolysis research.

Optimized pyrolysis conditions reported in the literature typically involve treatment at 500–650 °C for 30–60 min (in air or nitrogen atmospheres), achieving over 99% EVA removal and recovering high-purity glass, silicon, and metals (e.g., Ag, Cu) [73,74,76,77]. Furthermore, in an air atmosphere, the combustion process can be optimized by precisely controlling the equivalence ratio to minimize the generation of volatile organic compounds (VOCs) [74].

Currently, high energy consumption and potential gaseous emissions represent the primary challenges associated with the pyrolysis method. Future research efforts should focus on the development of low-energy clean combustion technologies and the integration of heat recovery systems.

#### 4.1.3. Thermal Cutting

Thermal cutting primarily utilizes heated tools such as hot knives or electric heating wires to soften the EVA, reducing its adhesive strength, thereby enabling the separation of the glass, cell layer, or backsheet. As early as 2016, NPC Inc. of Japan designed a dedicated hot-knife separation device for delaminating EOL PV modules. In this device, the hot knife is heated to 180–200 °C by an auxiliary heating unit. The module is conveyed via a drive roller group to the hot knife, where localized heating at the contact interface softens the EVA and weakens its adhesion, ultimately achieving separation of the glass from the PV cell layer [78]. Wahman et al. [79] further investigated the application of hot-knife technology for backsheet recovery from c-Si PV modules. Their method employs a hot air gun equipped with a precise temperature control system to heat an ultra-thin blade with high thermal conductivity, which softens the EVA to facilitate backsheet peeling. The study demonstrates that a blade temperature of 200 °C is optimal for achieving efficient, high-quality separation. Under this condition, the separation process requires no manual assistance, and the backsheet can be peeled off completely and intact in just 2.5 min. The purity of the recovered backsheet reaches as high as 99.42%, while the cells and the EVA layer remain undamaged.

Temperature control is a very important factor in thermal cutting technology. If the temperature is too low (below 150 °C), the adhesive is insufficiently softened, necessitating manual assistance and increasing the risk of damage to the backsheet and other layers, resulting in poor separation quality. Conversely, if the temperature is too high (exceeding 250 °C), although separation is accelerated, the EVA and backsheet may undergo thermal deformation or even melting, causing material accumulation on the blade and similarly compromising the separation outcome [79].

#### 4.1.4. High-Voltage Pulse Fragmentation

High-voltage pulse fragmentation utilizes high-voltage pulses to induce instantaneous discharge within or at the interfaces of materials, generating localized high temperature, high pressure, and shock waves. This enables “selective fragmentation” along the interfaces between different materials [80]. The fragmentation process involves the synergistic action of two mechanisms [81]: (a) electrical disintegration (ED), where high voltage causes breakdown within the solid material, leading to selective crushing at material boundaries; and (b) electro-hydraulic disintegration (EHD), where breakdown in the surrounding liquid solution generates shock waves that cause physical destruction of the material. Owing to its strong selectivity, the process yields high-purity silicon and facilitates metal recovery from EOL PV modules [82,83]. The relevant literature findings are summarized in Table 4.

As shown in Table 4, Nevala et al. [82] demonstrated that electro-hydraulic fragmentation achieves selective enrichment of metals in coarse fractions (>4 mm) and high-purity silicon (0.5–2 mm), whereas conventional crushing lacks such selectivity. Zhao et al. [83] further highlighted that while increasing voltage and pulse number raises the silver recovery rate, it lowers process selectivity; an appropriate electrode gap can improve silver enrichment without markedly sacrificing recovery. Song et al. [80,84] investigated the relationship between fragmentation performance and energy consumption, and identified the optimum conditions (160 kV, 300 pulses), which reduce energy consumption to roughly half that of mechanical crushing and achieve efficient metal concentration, though industrial implementation is still limited by equipment and scale-up challenges.

#### 4.1.5. Solvent Approaches

Solvent-based methods have become a widely studied area in c-Si PV module delamination research in recent years. Their fundamental principle involves using organic solvents or solvent systems to swell [85,86,87,88,89,90], dissolve [85,90,91,92], or chemically degrade [93,94,95,96,97] the polymer encapsulant layer (primarily EVA) within PV modules, thereby weakening the interfacial adhesion between the encapsulant and the glass, solar cells, and backsheet, achieving low-damage or non-destructive separation of each component. Compared with traditional mechanical crushing or pyrolysis methods, solvent-based approaches enable delamination under relatively mild conditions, helping to preserve the integrity and purity of glass, silicon wafers, and metal electrodes, thus creating favorable conditions for subsequent high-value recycling. Table 5 summarizes the specific processes, parameters, and results of solvent-based PV module delamination reported in recent years.

The solvents currently used can be mainly classified into the following categories:

▪ Hydrocarbon solvents, e.g., hexane;
▪ Polar aprotic solvents, e.g., N,N-dimethylacetamide (DMAC), methyl-2-pyrrolidone (NMP), N,N′-dimethylpropenylurea (DMPU);
▪ Ester solvents, e.g., EGDA, DBE, DMC, IPM + DEM;
▪ Bio-based solvents, e.g., ethanol, limonene, terpinolene;
▪ Supercritical fluids, e.g., supercritical CO_2_, supercritical n-butanol;
▪ Deep eutectic solvents (DES), e.g., choline chloride-oxalic (ChCl-Oxa) acid DES, menthol-decanoic acid DES.

Based on the characteristics of the above solvents and the literature [85,86,87,88,89,90,91,92,93,94,95,96,97,98,99,100,101,102,103,104] summarized in Table 5, the following conclusions can be drawn:

Toxicity: Hexane and polar aprotic solvents (DMAC, NMP) exhibit high toxicity and pose challenges in waste liquid treatment. In contrast, ester solvents, supercritical fluids, bio-based solvents, and deep eutectic solvents (DES) are generally non-toxic or low-toxicity, with most of them classified as green solvents that are more environmentally friendly, and some are biodegradable or recyclable.

Processing conditions and duration: The majority of solvents operate at 55–175 °C with ultrasonic assistance (200–900 W), where heating and ultrasonication serve primarily to shorten processing time. DES and supercritical fluids do not require ultrasonic assistance. Regarding processing duration, most solvents complete delamination within 2 h, although some DES (e.g., choline chloride–oxalic acid type), hexane, and supercritical CO_2_ require several hours or longer.

Solar cell integrity: Hexane, NMP, DMPU, limonene, ChCl-Oxa DES, and the optimized IPM/DEM process can effectively preserve the integrity of silicon wafers.

Solvent recyclability: NMP, DMC, IPM/DEM, ethanol, terpinolene, supercritical fluids, and DES can be recovered and reused.

Degree of separation: The vast majority of solvent systems achieve complete delamination of glass, solar cells, and backsheet in a single step. Only a few methods, such as DMAC, DMC, IPM/DEM, and the ethanol-alkali approach, require multiple steps to achieve full delamination.

#### 4.1.6. Laser Peeling

Laser peeling is a rapidly developing advanced delamination technology. Its principle is to utilize a laser to induce localized heating or generate stress waves at the material surface or interface to precisely destroy the adhesive layer, thereby achieving the selective separation of multilayer structures [46]. This technology offers advantages such as non-contact operation, high controllability, minimal damage, and clean separation interfaces. Furthermore, it is easily integrated into automated processes [105,106]. In recent years, it has garnered widespread attention in the field of PV recycling. The latest advances and key process parameters of laser peeling for PV module delamination are summarized in Table 6.

Studies by Li et al. [105] and Anwar et al. [106] have shown that the effectiveness of laser peeling is highly dependent on the precise control of laser parameters. Excessive laser power density and pulse repetition rate can cause thermal decomposition and carbonization of the EVA, as well as damage to the back electrode. Conversely, insufficient parameters lead to inadequate interfacial weakening, requiring greater mechanical force and making the EVA film prone to deformation [105]. Furthermore, the longer the laser wavelength, the higher the energy density required to achieve separation [106].

#### 4.1.7. Other Methods

In addition to the methods discussed above, Table 6 summarizes several other delamination technologies reported in recent years, such as electrothermal pulse fragmentation, waterjet cutting, and hybrid approaches (such as laser combining with high-voltage pulse, cryogenic pretreatment combining with mechanical crushing).

Zhang et al. [107] proposed an innovative delamination technology named “electrothermal pulse.” This technique applies a capacitor discharge pulse lasting 1 s to the module under an argon atmosphere (optimal condition: 200 V with a heating rate of 3373 K/s), exploiting the differences in thermal expansion coefficients among the layer materials to instantaneously generate an extremely high-gradient thermal field. The drastic non-uniform thermal expansion and contraction induce thermal stresses at the interlayer interfaces, achieving ultra-rapid and clean delamination within 1 s. This method significantly suppresses the pyrolysis of organic materials, enabling the EVA film and TPT backsheet to be recovered as nearly intact films, which facilitates subsequent resource utilization.

According to Bellini’s report [111], Shintora Kosan Co., Ltd. in Japan has developed a novel water jet technology. This technology sprays high-pressure water from the back side of PV modules to peel off and crush the solar cells and backing materials without damaging the glass. After treatment, the glass surface is free of any harmful residues or damage.

Multi-technology synergistic processes also represent a promising direction for further development. For example, Ali et al. [112], through an analysis of various mechanical methods, concluded that no single method is sufficient to achieve comprehensive material recovery. Based on data analysis, they proposed a hybrid approach combining laser and high-voltage pulse technologies: first, laser irradiation is used to remove the EVA encapsulant, followed by high-voltage pulse fragmentation to selectively decompose and separate the remaining components. However, this method has not yet been experimentally validated. In addition, utilizing the principle that the brittleness of the EVA encapsulant, backsheet, and glass in photovoltaic modules increases significantly at low temperatures, scholars such as Zhang et al. [108], Wei et al. [109], and Wu et al. [110] have, respectively, proposed a treatment method combining cryogenic pretreatment with mechanical crushing. This method involves first freezing waste PV modules using liquid nitrogen or similar methods, followed by mechanical crushing and screening to separate and recover the components. Furthermore, the combination of thermal cutting and solvent-based methods is expected to improve delamination efficiency while ensuring the integrity of the glass and solar cells.

### 4.2. Silicon and Valuable Metals Recovery

#### 4.2.1. Recovery and Purification of Silicon from c-Si PV Cells

To enable the high-value use of silicon recovered from EOL c-Si PV modules, detached solar cells must be purified to remove impurity layers, including Ag grid electrodes, antireflective (AR) coatings, n-p junctions, and Al back electrodes. Chemical etching is the dominant process for this purpose. Its effectiveness and the difficulty of subsequent processing critically depend on parameters including the etching solution composition, temperature, duration, and sequence. Depending on the required purity of the reclaimed silicon and its intended application, existing processes can be broadly categorized into two types, with the relevant techniques and key parameters summarized in Table 7.

The first category of processes focuses on removing the silver and aluminum electrodes from the solar cell surface through relatively simple etching steps. This yields silicon that, while falling short of solar-grade purity, can be directly utilized in other applications (e.g., lithium-ion battery anodes). For example, Wang et al. [113] compared the efficacy of HNO_3_ and HCl solutions, combined with ultrasonic assistance, in selectively removing the Ag grid lines and Al back electrodes, respectively. Chen et al. [114] employed a two-step etching method, first with HNO_3_ and subsequently with KOH solution to leach out the Ag and Al electrodes. Bao et al. [59] treated the recovered silicon with a 3 wt% NaOH solution and composited it with banana peel bio-carbon to fabricate a silicon-carbon anode for lithium-ion batteries (LiBs). This anode delivered a capacity of 1378.60 mAh/g after 500 cycles at 200 mA/g. Similarly, Eshraghi et al. [60] sequentially used KOH and HNO_3_ to remove Al, Ag, and Pb, processing the silicon into a nanostructured material. This material meets the requirements for an expansion-tolerant silicon anode in LiBs, retaining a capacity of approximately 1400 mAh/g after 50 cycles.

The second category of processes aims for the deep chemical purification of spent solar cells. Through stringent etching steps, this approach completely removes the Ag electrodes, AR coating, p-n junction, and Al back electrode, yielding high-purity silicon suitable for direct reuse in manufacturing new solar cells. HF is commonly employed to remove the AR coating and p-n junction due to its effectiveness in dissolving silicon nitride and silicon dioxide. For instance, Huang et al. [76] sequentially etched the solar cells with HNO_3_/HF/NaOH solutions, ultimately achieving over 90% recovery of solar-grade silicon. Klugmann-Radziemska and Ostrowski [12] recommended initially removing the Al layer with KOH, followed by a mixed acid (HNO_3_/HF/CH_3_COOH/Br_2_) to eliminate the Ag electrodes, AR coating, and p-n junction, while emphasizing that etching formulations must be tailored to specific cell types. Kang et al. [115] utilized a mixed acid of HF/HNO_3_/H_2_SO_4_/CH_3_COOH/H_2_O at room temperature and obtained silicon with a purity of 99.999% and a recovery rate of 79%. Notably, they highlighted that adding 20 wt% surfactant to the mixed-acid solution can increase the silicon recovery rate to 86%. Park et al. [116] compared two different processes: Process-1 involved front-side etching with an HNO_3_/HF mixture followed by back-side KOH treatment. However, excessive etching resulted in overly thin wafers. In Process-2, the Al electrode and silicon nitride layer were first removed using H_3_PO_4_ at 160 °C, followed by HNO_3_ and HF to eliminate the Ag electrodes, emitter, and p-n junction, yielding wafers with performance nearly identical to commercial virgin silicon. Li et al. [117] developed a purification process based on Al-Si solvent refining. This method first removes the Ag and Al electrodes with hydrochloric acid, then uses an HF solution to strip the AR coating, and finally eliminates boron and phosphorus impurities via segregation in an Al-Si melt. However, the authors noted that a secondary refining step is still required to meet solar-grade standards. Xu et al. [57] successively removed the Al and Ag electrodes along with the silicon nitride AR layer using HCl/HNO_3_/HF solutions. They subsequently applied a metal-assisted chemical etching (MACE) process to simultaneously remove residual phosphorus and aluminum impurities while constructing a nanostructured surface with ultra-low reflectivity.

Despite its excellent etching performance, HF poses high toxicity, severe corrosiveness, and risks to human health and the environment, requiring specialized equipment and introducing safety and environmental concerns in practice. Consequently, researchers are actively exploring alternative strategies. Chemically, phosphoric acid has been investigated as a substitute for HF to remove the silicon nitride AR coating and the p-n junction [30,118,119]. For example, Punathil et al. [120] initially used NaOH and HNO_3_ to remove the Al back-surface field and Ag grid lines, and subsequently applied a heated H_3_PO_4_ solution to eliminate the remaining AR coating and p-n junction, ultimately achieving a silicon purity of 99.9984%. Physically, Park et al. [121] applied mechanical grinding to directly remove the AR coating, significantly reducing chemical reagent consumption. Furthermore, Gao et al. [122] proposed an innovative process based on molten-salt etching. This method first employs a NaOH-KOH molten salt mixture at 200 °C to rapidly etch the solar cell surface coatings, liberating the Ag grid lines; the Al back layer is then removed using a NaOH solution. This approach not only achieves rapid, efficient, and high-purity silicon recovery but also entirely eliminates the need for toxic chemicals and harmful emissions throughout the entire process.

Currently, recovering silicon wafers from waste PV modules still faces several challenges, including the complexity and cost of recycling processes, compatibility issues due to differences across generations of cell technologies, a lack of standardized recycling protocols, and secondary pollution generated during treatment. Continued efforts are therefore needed to address these barriers and support the scalable and sustainable recycling of EOL c-Si PV modules.

#### 4.2.2. Methods of Recycling Valuable Metals

EOL c-Si PV modules contain high-value metals such as Ag, Cu, and Al; recovering these metals is crucial to prevent resource waste. Currently, the primary separation and recovery methods include physical crushing and screening (e.g., mechanical and high-voltage pulse crushing), laser ablation, and hydrometallurgy. For instance, Zhao et al. [83] applied high-voltage pulse crushing to fragment EOL PV modules, followed by screening and centrifugal concentration, ultimately achieving high recovery rates of 93.78% for Ag and 94.22% for Cu. Similarly, Lim et al. [123] employed a high-voltage pulsed wire explosion to selectively remove the Ag grid lines via electrical explosion, yielding a 69% Ag recovery rate.

Hydrometallurgy, characterized by high recovery rates, strong selectivity, mild reaction conditions, and flexible multi-metal recovery, has become the predominant method for reclaiming valuable metals from EOL c-Si PV modules. Given its highest economic value among the metals in EOL PV modules, Ag is the primary target of hydrometallurgical recovery. Currently, HNO_3_ is widely used to leach Ag from solar cells [30,76,114,124]. The dissolved silver is subsequently precipitated as silver chloride using chloride sources (e.g., NaCl or HCl), converted to silver oxide with NaOH, and finally reduced to metallic silver using reducing agents such as hydrazine hydrate (N_2_H_4_·H_2_O) [30] or glucose [114]. To achieve higher purity, the reduced silver can undergo further electrolytic refining. Alternatively, Huang et al. [76] developed a streamlined electrochemical route, directly depositing silver with a purity exceeding 99% from the nitric acid leachate via electrodeposition, thereby bypassing the multiple precipitation and conversion steps.

To eliminate nitrogen oxide emissions associated with nitric acid use, researchers have developed various eco-friendly and efficient alternative processes for silver recovery. Yang et al. [125] employed an eco-friendly solvent mixture of methanesulfonic acid (MSA) and hydrogen peroxide (H_2_O_2_) for room-temperature silver leaching. Subsequent precipitation, chemical conversion, and electrorefining yielded silver with a purity of up to 99.995%. Modrzynski et al. [126] proposed an electrochemically assisted leaching process using a boron-doped diamond electrode and sulfuric acid, followed by potential-controlled electrodeposition, achieving an overall recovery rate of 88%. Lemoine et al. [127] utilized the green deep eutectic solvent Ethaline (choline chloride-ethylene glycol) combined with iron(III) chloride hexahydrate (FeCl_3_·6H_2_O) to leach silver, achieving an efficiency exceeding 99.9%. Gao et al. [122] introduced a NaOH-KOH molten-salt etching method, enabling rapid etching of various crystalline silicon solar cells within 1–3 min. This process facilitated efficient silver separation and recovery (99.0%) while avoiding toxic chemicals and harmful emissions. Chung et al. [128] used an iodine-potassium iodide (I_2_-KI) solution to selectively leach 95% of the silver and aluminum within 5 min, subsequently separating the two metals via pH adjustment. Alsulami et al. [129] first dissolved the aluminum layer with NaOH, then used an FeCl_3_ solution to induce the surface oxidation of the silver grid lines, causing them to physically detach and yielding a 93% silver recovery rate. Table 8 summarizes the process flows and key parameters employed by various researchers using this approach.

Aluminum in EOL c-Si PV modules exists primarily in two components: the Al frame and the Al back electrode, with recovery methods varying based on their form and location. The Al frame can typically be recovered directly via mechanical disassembly. In contrast, the Al back electrode within the solar cells is mainly processed chemically, typically leached using alkaline solutions such as NaOH or KOH [9,30,60,119] to yield sodium or potassium aluminate. These intermediates can be directly utilized as industrial raw materials in sectors like papermaking or water treatment [130]. Alternatively, they can be precipitated as aluminum hydroxide via pH adjustment and subsequently calcined to produce alumina. Copper and tin in PV modules reside primarily in the solder ribbons. These metals can be recovered through hydrometallurgical routes coupled with electrodeposition [30,76,126], while copper can also be reclaimed via mechanical methods such as polishing [114].

## 5. Challenges and Prospects

### 5.1. Challenges of Recycling EOL c-Si PV Modules

**Figure 7:**
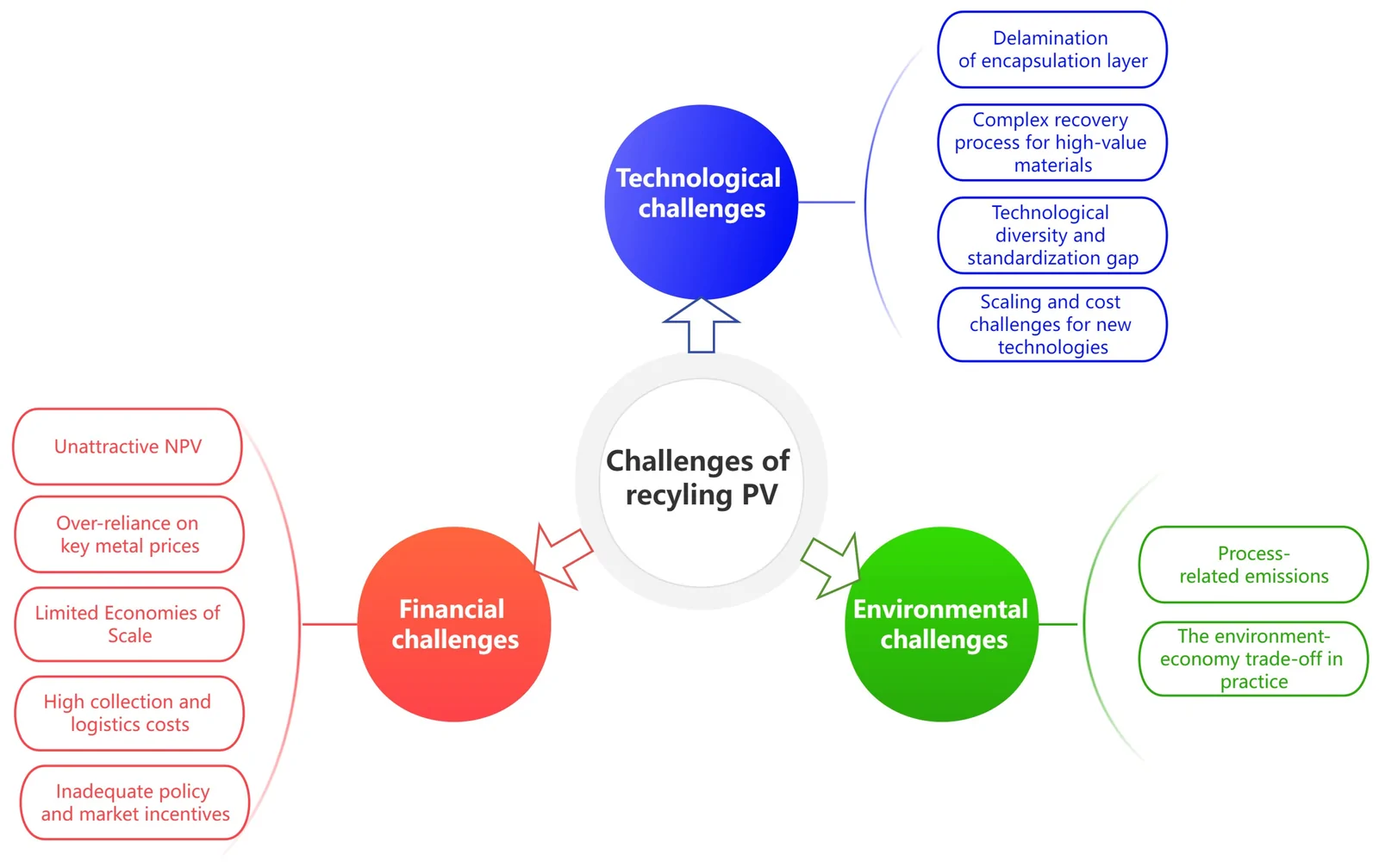
Technical, financial, and environmental challenges in the recycling of EOL c-Si PV modules.

Despite years of research yielding diverse recovery methods and processes for EOL PV modules, a significant gap remains before full-scale commercialization can be achieved. This gap stems from persistent challenges across technical, financial, and environmental dimensions, as illustrated in Figure 7.

#### 5.1.1. Technological Challenges

① Delamination of Encapsulation Layer (EVA):

The sealed laminate “sandwich” structure of PV modules, designed for durability, poses a major disassembly challenge during recycling. The core difficulty lies in removing the EVA encapsulant to separate materials such as glass and solar cells. Current mainstream methods all suffer from inherent limitations:

- Physical Treatment (Crushing): Methods like mechanical crushing, grinding, and high-voltage pulse fragmentation often cause brittle silicon wafers and valuable materials like silver grid lines to fragment and intermix with other components, hindering efficient separation and purification. This leads to significant material loss and diminishes the recovery value of silicon [105,107]. While high-voltage pulse crushing offers more precise separation, it involves high equipment costs, substantial energy consumption, and limited throughput.
- Thermal Treatment: Pyrolysis effectively decomposes EVA, but it is energy-intensive and may compromise material purity. High temperatures can oxidize metals (e.g., silver, copper) or cause silicon wafers to fracture, reducing both recovery rates and reuse value. Additionally, the thermal decomposition of EVA and backsheet materials (e.g., Tedlar) releases toxic gases [131,132]. Thermal cutting and laser peeling methods allow for efficient and non-destructive recovery. However, their processing rates are limited by tool paths, scanning speeds, and heat transfer efficiency, making it difficult to achieve continuous high-throughput operation [16].
- Chemical Treatment: Processing times are typically prolonged. Furthermore, there is a significant trade-off: conventional solvents are highly toxic, whereas green alternatives are generally expensive. Effluent treatment remains challenging and poses a risk of secondary pollution. Moreover, immersion in certain solvents will cause silicon cells to fracture due to swelling pressure [133,134].

② Complex Processes for High-value Materials:

Current industrial practices still focus mainly on “bulk recycling,” involving simple crushing and sorting in existing glass/metal recovery facilities. This approach primarily recovers glass, aluminum frames, and copper wires, yet fails to effectively recover the most valuable components like silicon wafers and silver. Silicon wafers are frequently downcycled into low-value silicon powder. Although laboratory-developed “high-value recovery” technologies can reclaim silicon wafers and precious metals, most processes are overly complex (involving multi-step chemical etching and metallurgical treatments) and have not yet been scaled up for commercial application.

③ Technological Diversity and Standardization Gaps:

PV modules vary significantly in size, material composition, and structure depending on the type. Furthermore, advancements in cell technology have altered the material makeup of PV modules, potentially requiring tailored recycling processes. This heterogeneity complicates the development of recycling facilities and makes it difficult to establish unified, efficient, and standardized processes capable of handling multiple technology generations of modules [135,136].

④ Scaling and Cost Challenges for New Technologies:

Although innovative technologies such as high-voltage pulse crushing, laser ablation, supercritical fluids, and green solvents have been developed in laboratories in recent years, aiming to improve material recovery rates, purity, and value in an eco-friendly manner, most have not yet achieved stable commercial-scale application. Their equipment and processing costs, energy consumption, and throughput efficiency still require further optimization.

#### 5.1.2. Financial Challenges

① Unattractive Net Present Value (NPV):

High recycling costs remain a primary barrier to the PV recycling industry. The process encompasses multiple stages: collection, transportation, dismantling, material separation, purification, and waste treatment, each incurring substantial expenses. Conversely, the market value of the primary recovered materials (e.g., cullet glass, scrap metals) is generally low, often failing to offset total costs. Multiple studies [26,137,138,139] indicate that under current industrial conditions, the NPV of PV recycling projects is frequently negative, highlighting their lack of economic viability.

② Over-reliance on Key Metal Prices:

Economic feasibility is highly sensitive to the recovery rate and market prices of high-value materials, particularly silver and intact silicon wafers. Silver recovery is a critical determinant of project profitability [137,139]. However, silver content in PV modules is minimal (about 0.1%) and is projected to decline further with technological advancements. Additionally, the complexity of efficient silver extraction renders the economic model fragile and highly vulnerable to commodity price fluctuations.

③ Limited Economies of Scale:

Although future volumes of decommissioned modules are projected to be massive, the current influx of EOL PV modules in most countries has not yet formed a stable, large-scale waste stream. This leads to underutilized capacity at dedicated recycling facilities, elevated unit-processing costs, and difficulties in attracting large-scale commercial investment. According to [26], a plant must process at least 16,000 tons/year of PV waste to be economically viable.

④ High Collection and Logistics Costs:

PV installations are typically geographically dispersed and often situated in remote areas. Consequently, the costs associated with collecting decommissioned modules and transporting them to centralized processing facilities are substantial, further undermining the economic feasibility of recycling.

⑤ Inadequate Policy and Market Incentives:

In many major PV-deploying countries, mandatory PV waste recycling regulations, extended producer responsibility (EPR) schemes, and effective financial incentives (e.g., subsidies or tax benefits) remain either inadequate or poorly enforced. Without policy drivers and the internalization of external costs, recycling cannot economically compete with low-cost disposal methods like landfilling [4].

#### 5.1.3. Environmental Challenges

① Process-related Emissions:

Recycling processes inherently carry an environmental footprint. Pyrolysis is energy-intensive and may generate greenhouse gas emissions [140], while the thermal degradation of EVA polymers can release hazardous volatile compounds such as acetic acid, propene, and 1-hexene [121]. Chemical treatments involve hazardous reagents and byproducts that risk causing secondary pollution if improperly managed [21,33]. Even mechanical crushing generates dust and noise. Consequently, recycling operations must be systematically optimized to minimize their energy consumption and direct emissions.

② The Environment-economy Trade-off in Practice:

From a Life Cycle Assessment perspective, closed-loop recovery of high-value materials, particularly silicon and silver, offers the greatest potential to offset the substantial environmental burdens (e.g., energy consumption, carbon emissions, and toxic impacts) associated with raw material extraction and refining [141], representing the environmentally optimal choice. However, realizing “full recovery” currently faces significant technical and cost challenges [121,142]. Conversely, the “bulk-recovery” approach is technologically mature and economically viable, yet its environmental benefits remain marginal due to the loss of high-value materials.

### 5.2. Prospects

Although significant progress has been made in the field of EOL PV module recycling, achieving large-scale commercial application still requires coordinated efforts across multiple dimensions, including technology, management, policy, and market mechanisms.

(1) Technology Breakthrough

Future research should focus on efficient, low-loss, green, and cost-controllable delamination and purification processes. For delamination, efforts could focus on technologies such as recyclable green solvents, laser peeling, or electrothermal pulse peeling to enable high-value recovery of glass, silicon wafers, and metal electrodes. Multi-technology synergistic processes, such as “laser pretreatment + high-voltage pulse fragmentation” or “thermal cutting + green solvents,” could also be explored to improve processing efficiency. In terms of purification, the industrialization of fluorine-free, low-toxicity chemical etching alternatives (e.g., phosphoric acid instead of hydrofluoric acid, salt-etching) and electrochemical deposition should be promoted to reduce the risk of secondary pollution.

(2) Management Optimization

A digital lifecycle management system for PV modules should be established, promoting the adoption of “electronic IDs” or QR code labels to record information such as production batch, material composition, installation year, and maintenance history, thereby facilitating sorted disposal and cascade utilization at decommissioning. Meanwhile, consideration may be given to building regional collection, transportation, and temporary storage networks, or establishing a hierarchical network featuring “distributed pretreatment + centralized purification” to reduce logistics costs and improve recycling efficiency.

(3) Policy and Regulation Improvement

Countries should accelerate the formulation or improvement of dedicated regulations for EOL PV modules, clarifying the implementation rules of Extended Producer Responsibility to ensure that manufacturers assume full-chain responsibility from collection to final disposal. Unified recycling rate accounting standards and certification systems should also be established to regulate the access qualifications of recycling enterprises. It is recommended that PV modules be included in specific category waste management lists to strengthen the safe disposal supervision of lead-containing and fluorine-containing materials. At the international level, it is advisable to advance the development of globally unified recycling technology standards and data-sharing, thereby facilitating cross-border cooperation and technology transfer.

(4) Subsidy and Market Incentive Mechanisms

Economic feasibility remains the primary bottleneck constraining the industrialization of PV recycling. It is recommended that governments establish special funds or green credit schemes, providing equipment subsidies or tax reductions for enterprises adopting advanced green recycling technologies. Recycled materials should receive green product certification and be prioritized for inclusion in government procurement. Furthermore, a “recycling quota trading mechanism” could be explored, whereby producers failing to meet their recycling obligations are charged a regulatory fee, which is then used to subsidize compliant enterprises, thereby forming a closed-loop market-based incentive system.

## 6. Conclusions

With the advent of the global wave of EOL c-Si photovoltaics, achieving green disposal and high-value recycling of c-Si PV modules is of paramount importance. This paper systematically reviews the technological pathways for module delamination, silicon purification, and metal recovery, and analyzes the current challenges.

The review shows that c-Si PV recycling technologies are moving from conventional mechanical crushing and pyrolysis toward greener, more precise methods. Conventional processes are technologically mature but suffer from high material loss, high energy consumption, and risks of secondary pollution. In contrast, certain low-toxicity solvents (such as NMP), green solvents including deep eutectic solvents and bio-based solvents, and novel delamination techniques such as laser peeling and electrothermal pulse peeling can achieve non-destructive or low-damage separation of glass, silicon wafers, and metal electrodes.

Progress has also been made in purification and metal recovery using green technologies. In silicon purification, salt-etching methods such as molten NaOH-KOH can remove surface coatings within 1–3 min, avoiding the safety and environmental risks of hydrofluoric acid. In metal recovery, technologies combining deep eutectic solvents or salt-etching with electrodeposition can replace traditional hydrometallurgy, recovering high-purity silver and copper from leach solutions with high selectivity and low energy consumption, while avoiding the chlorine-containing wastewater problem of conventional chemical precipitation. These green technologies are expected to play a larger role in the future of PV recycling.

For future research, the following directions are recommended: (1) developing recyclable green solvents, laser peeling, and electrothermal pulse peeling, while exploring combined processes such as “laser pretreatment + high-voltage pulse fragmentation” or “thermal cutting + green solvents” to improve processing efficiency and material recovery rates; (2) scaling up fluorine-free chemical etching (e.g., salt-etching, phosphoric acid substitution for hydrofluoric acid) and recyclable green solvents to reduce secondary pollution; (3) building a digital lifecycle management system based on electronic identifiers, and optimizing a regional recycling network with “distributed pretreatment + centralized purification” to lower logistics costs; and (4) improving the EPR system and recycling quota trading mechanisms to create a market-based incentive loop and address the economic feasibility challenge.

## Figures and Tables

**Table 1:** Weight percentage of each component in a c-Si PV module.

Material Proportion in wt%			Ref. [14]	Ref. [15]	Ref. [16]
With recycling value	Metals	Silver	0.05	0.006~0.08	<0.08
		Aluminum frame and conductor	18.53	10.00~20.00	15.50~20.60
		Cables	1.00	-	1.00~1.20
		Copper	0.11	4.40~7.00	0.10~0.15
		Junction boxes (containing copper)	-	2.00	-
	Others	Si	3.65	2.00~3.00	2.00~4.00
		Glass	70.00	69.00~75.00	69.00~76.00
Without recycling value	Tin and lead		0.05	<0.20	<0.08
	Organic polymer materials (EVA and backsheet, etc.)		6.60	7.00	6.00~10.00

**Table 2:** Process steps and results of mechanical crushing for c-Si PV module treatment.

Process and Key Parameters	Recycled Products	Key Points	Year/Reference
Two-blade rotor crushing, Hammer crushing. Sieving, Pyrolysis (particles > 1 mm), 650 °C, 1 h.	Glass	✧ Glass recovery is 85%, ✧ Particles (d < 0.08 mm) require further processing.	2014/[65]
Hydraulic shear, Chain crushing.	Glass, silicon and metals	✧ Metal recovery is 60%.	2017/[63]
Double rotor crushing (triple broken), Sieving, Pyrolysis (particles > 1 mm), 650 °C, 1 h, Acid leaching (0.08 mm < particles < 0.4 mm).	Glass	✧ Glass recovery is 91%.	2017/[66]
Mechanical milling, Electronic separation.	Glass, silicon, and metals	✧ Metals can be enriched, ✧ This process fails to effectively separate polymers.	2018/[68]
Shredded with a knife mill, Heavy medium separation, Milling, 45 s, Sieving.	Glass and valuable metals	✧ Glass recovery is 76% (100% grade), ✧ Metals recovery is 100% (67.2% grade).	2019/[64]
Shear-press machine, Swinging hammers mill, Sieving, Fixed hammers mill, Three-stage vibrating screen.	Glass, silicon and metals	✧ A mobile mechanical treatment system was developed.	2019/[69]
Shredding, Gravity separation + sieving, Eccentric stirring milling, Selective grinding, 5 min.	Glass	✧ 97% of the glass was concentrated into particles < 5.6 mm.	2021/[56]
A specifically designed single-shaft shredder, Sieving, Solvent treatment, cyclohexane, 1.5 h.	Glass and valuable metals	✧ Coarse particles contain the most valuable components.	2021/[70]
Crushing by a cutting mill, Three crushing–screening cycles.	Silver	✧ Ag is highly concentrated in the particle fraction < 0.25 mm.	2023/[67]

**Table 3:** Process steps and results of pyrolysis for c-Si PV module treatment.

Process and Key Parameters	Recycled Products	Key Points	Year/References
Heating (170 °C, 10 min), manually peel backsheet Pyrolysis, 600 °C, 33 min, air atmosphere.	Glass, silicon cell, and welding ribbon	✧ Increasing the temperature linearly reduces the processing time, ✧ Backsheet pre-removal reduces processing time, harmful gas emissions, and solid residues.	2017/[76]
Manually peel backsheet, Mechanical crushing, Sieving, Pyrolysis, 500 °C, 30 min, nitrogen atmosphere.	Silicon and metals (Ag, Cu)	✧ More than 99% of polymers (EVA) can be removed.	2017/[77]
Heating (150 °C, 5 min), manually peel backsheet, Pyrolysis, 500 °C, 1 h, nitrogen atmosphere.	Glass and silicon cell	✧ The pyrolysis products of EVA are mainly acetic acid and hydrocarbons.	2019/[73]
Heating (200 °C, 450 °C/h, 30 min), manually peel backsheet, Pyrolysis, 500 °C, 450 °C/h, 1 h, slightly oxidizing atmosphere.	Glass, silicon, and metals (Ag, Cu, Al)	✧ VOC emissions can be controlled, ✧ Avoid fluorinated gas emissions.	2019/[74]
Mechanical milling for backsheet removal, Pyrolysis, 500 °C, 450 °C/h, 1 h, oxidizing atmosphere.	Glass, silicon, and metals (Ag, Cu, Al)	✧ Avoid fluorinated gas emissions, ✧ Optimize processing energy consumption.	2019/[75]
Halogen lamp heating (90–120 °C), manually peel backsheet, Pyrolysis, 650 °C, 30 min, air atmosphere, Acid etching.	Silicon and metals (Ag, Cu, Pb)	✧ Silicon recovery rate is about 89–94%.	2021/[72]

**Table 4:** High-voltage pulse fragmentation for c-Si PV module delamination.

Process and Key Parameters	Recycled Products	Key Points	Year/Reference
Electro-hydraulic fragmentation, 300 pulses. Sieving.	Silicon and metals	✧ Most metals are enriched in the particle fraction >4 mm (Cu ~99%, Ag ~60%, Pb/Sn/Al ~80%), ✧ The purity of silicon exceeds 99% in the particle fraction (0.5–2 mm).	2019/[82]
High-voltage pulse crushing. Gravity sorting/Eddy current sorting.	Glass and silver	✧ Crushing selectivity: Ag > Si > glass, ✧ Increasing the voltage and pulse number can increase the silver recovery rate but decreases the enrichment rate.	2020/[83]
High voltage fragmentation, 160 kV, 300 pulses. Sieving.	Glass, silicon, and metals (Ag, Al, Cu)	✧ The energy consumption is approximately half that of conventional mechanical crushing, ✧ Industrial-scale production is highly challenging.	2020/[80,84]

**Table 5:** Solvent methods for c-Si PV module delamination.

Solvent Type	Solvent	Process and Key Parameters	Key Points	Year/Reference
Hydrocarbon solvents	Hexane	6.1 mol/L (concentration), 50 g/L (solid-to-liquid ratio), 73 °C, Ultrasonic (250 W), 24 h	✧ Wafer cells without significant damage, ✧ Ultrasonic treatment can enhance separation efficiency.	2026/[98]
Polar aprotic solvents	N,N′-Dimethylpropenylurea (DMPU)	1/12 g/mL (solid-to-liquid ratio), 160 °C, Ultrasonic (900 W), 10 min	✧ Wafer cells remain intact, ✧ Short processing times, ✧ Higher ultrasonic power reduces the separation time.	2022/[91]
	N,N-Dimethylacetamide (DMAC)	Fluorinated coating separation: 25 °C, 5 min Glass separation: 160 °C, 10 min Remaining components: Ultrasonic (800 W, 20 Hz), 10 min	✧ Short processing times, ✧ Heating and ultrasonic treatment are crucial for the delamination process.	2025/[92]
	methyl-2-pyrrolidone (NMP)	170 °C, 30 min	✧ Solvents can be recycled and reused, ✧ The PV laminated module is completely decapsulated, ✧ Low toxicity, high boiling point, and relatively low cost.	2025/[85,99]
Ester solvents	Ethylene glycol diacetate (EGDA)	2 × 2 cm, 1/15 g/mL (solid-to-liquid ratio), 130 °C, Ultrasonic (900 W, Intermittent operation), 30 min	✧ Good wettability on glass, ✧ Increasing temperature and ultrasonic power could accelerate the glass separation, ✧ Heat treatment is still required to obtain clean wafer cells.	2023/[86]
	Dibasic ester (DBE)	2 × 2 cm, 1/15 g/mL (solid-to-liquid ratio), 140 °C, Ultrasonic (900 W), 30 min	✧ Processing times increase with increasing fragment size, ✧ Heat treatment is still required to obtain clean wafer cells.	2023/[87]
	Dimethyl carbonate (DMC)	2 × 2 cm, 1/6 g/mL (solid-to-liquid ratio), 80 °C, 60 min (glass), 90 min (backsheet)	✧ Solvents can be recycled and reused, ✧ Heat treatment is still required to obtain clean wafer cells.	2025/[93]
	Isopropyl myristate (IPM) + diethyl malonate (DEM)	2 × 2 cm, Ultrasonic (800 W), 140 °C, IPM treatment (15 min), DEM treatment (45 min)	✧ Solvents can be recycled and reused, ✧ IPM and DEM exhibit a strong wetting effect on glass and EVA, ✧ Wafer cells remain intact with the optimized process.	2025/[100]
Bio-based solvents	Ethanol	5 × 5 cm, Ethanol (75%), 6/25 g/mL (solid-to-liquid ratio), 70 °C, 30 min	✧ Achieved complete backsheet separation, ✧ Ethanol can be reused.	2024/[94]
	Anhydrous ethanol + aqueous sodium carbonate solution	Mechanical removal of glass: 170 °C, 5 min Aluminum back electrode and back EVA film separation: Anhydrous ethanol, 200 °C, 15 min Silver grid lines and the front EVA: Na_2_CO_3_ (0.3 mol/L), 200 °C, 120 min	✧ Pre-removal of glass, ✧ The EVA film is completely removed, ✧ Environmentally safe.	2025/[101]
	Limonene	2 × 2 cm, Limonene (0.1 M), 1/15 g/mL (solid-to-liquid ratio), 55 °C, Ultrasonic (800 W), 20 min	✧ Wafer cells remain intact, ✧ Achieves complete separation of the glass, solar cells, and backsheet, ✧ The solvent is non-toxic and biodegradable.	2024/[88]
	Terpinolene	2 cm × 2 cm, 90 wt% terpinolene, 80 °C, Ultrasonic (800 W), 55 min	✧ Achieve complete separation of the glass, solar cells, and backsheet, ✧ Solvents can be recycled and reused.	2026/[102]
	Sunflower oil + enzyme	2.5 × 2.5 cm, 0.1–0.61% (*v*/*v*) Enzyme (lipase, laccase, or lecitase), 25.0–44.83%(*v*/*v*) sunflower oil, 30–60 °C, 0.1 M phosphate buffer, pH 6, 72 h	✧ Delamination rate of 100%, ✧ Environmentally safe, ✧ Lower processing temperature.	2025/[95]
Supercritical fluids	Supercritical CO_2_	POE: ~60 °C, ~100 bar, 6 h EVA: ~75 °C, ~140 bar, 6 h EMAA: ~90 °C, ~120 bar, 6 h Depressurization rate: 1.7 bar·s^−1^	✧ The processing efficiency depends on the affinity of the encapsulating material for CO_2_, ✧ Delamination effect: EVA > EMAA > POE.	2022/[89] 2023/[103]
	Supercritical n-butanol	6 cm × 1.5 cm, 470 mL, 328 °C, N_2_ atmosphere, 43 min	✧ Chemical degradation occurs, ✧ Clean cell surfaces are obtained.	2025/[104]
Deep eutectic solvents (DES)	Choline chloride + oxalic acid DES	Synthesized in 1:1 molar ratio, 2.0 × 2.0 cm, 175 °C, 12.5 h	✧ The EVA separation rate reached 100%, ✧ Environmentally safe, ✧ Solvents can be recycled and reused, ✧ Long processing times.	2024/[96]
	Choline chloride + oxalic acid dihydrate DES	Synthesized in 1:1 molar ratio, 175 °C, 7.5 h	✧ The EVA separation rate reached 100%, ✧ Environmentally safe, ✧ Lower viscosity and excellent wettability.	2025/[97]
	Menthol + decanoic acid DES	Synthesized in 1:1 molar ratio, 80 °C, 2 h	✧ Extremely high dissolution capability for EVA, ✧ Lower processing temperature, ✧ The solvent has switchable hydrophilicity, enabling easy recovery and reuse.	2024/[90]

**Table 6:** Laser peeling and other methods for c-Si PV module delamination.

Technology	Process and Key Parameters	Recycled Products	Key Points	Year/Reference
Laser peeling	1064 nm near-infrared pulsed fiber laser (2.1 MW/cm^2^, 50 kHz) + mechanical peeling.	EVA and silicon cell, glass	✧ EVA layer completely and purely peeled off, ✧ Solar cell structure undamaged, ✧ Peeling effect strongly dependent on power density and pulse repetition rate.	2022/[105]
	Nanosecond pulsed laser peeling (355 nm, 0.23 J/cm^2^).	Glass and silicon cell	✧ The main structure of the solar cell remains intact, ✧ The surface anti-reflective layer sustains damage, ✧ glass-EVA layer separates automatically under gravity.	2024/[106]
Electrothermal Pulse peeling	Electrothermal Pulse (200 V, 1 s, Thermal gradient: 3373 K/s).	Glass and silicon cell	✧ Glass recovery is 100%, ✧ The recycling rate of silicon solar cells is 98.22%, ✧ The voltage significantly influences the peeling effect.	2025/[107]
Cryogenic pretreatment + mechanical crushing	Liquid nitrogen cryogenic modification, 5 min, Three-blade rotor crushing, 8 s, Mechanical screening.	Glass and silicon	✧ The silicon enrichment rate reached a maximum of 72%, ✧ The glass removal rate was 70%.	2021/[108]
	Angle grinder cutting, Cryogenic freezing, Jaw crushing.	glass	✧ The glass removal rate reached a maximum of 99.6%.	2024/[109]
	Liquid nitrogen cryogenic modification, 3 min, Shredding, Pyrolysis, 600 °C, 1 h, argon atmosphere, Mechanical screening.	Aluminum and silicon	✧ Silicon recovery is 88.2% (86.1% purity), ✧ Al recovery is 86.0% (100% purity).	2023/[110]

**Table 7:** Process and parameters for silicon recovery from c-Si PV cells.

Processing Objective	Process and Key Parameters	Key Points	Year/References
Simple treatment: Removing the Al coating and Ag electrodes	Method 1: 60 °C, HCl (3 mol/L), Ultrasonic (150 W), 2 h, Method 2: 60 °C, HNO_3_ (3 mol/L), Ultrasonic (150 W), 1.5 h.	✧ Method 1: The recovery rate of silicon is 99.24% (99.85% purity), ✧ Method 2: The recovery rate of silicon is 98.9% (99.78% purity).	2022/[113]
	80 °C, HNO_3_ (5 mol/L), 25 mL/g, 1 h, 80 °C, KOH (1 mol/L), 50 mL/g, 1 h.	✧ The recovery purity of Si is 99.84%; the recovery rate is 99.9%.	2021/[114]
	NaOH (3 wt%).	✧ Recovered silicon can be used together with waste banana peels to prepare silicon-carbon anodes for LIBs, ✧ Providing 1378.60 mAh/g capacity after 500 cycles.	2025/[59]
	60 °C, KOH (8 mol/L), 5 min, 80 °C, HNO_3_ (8 mol/L), 15 min.	✧ Recovered Si meets the requirements for expansion-tolerant silicon anodes in LIBs, ✧ Providing 1400 mAh/g capacity after 50 cycles.	2020/[60]
Deep treatment: Removing the Al coating, Ag electrodes, AR Layer, and n-p junctions	HNO_3_ (30%), HF (10%), 15 min, 50 °C, NaOH (3%), 30 min.	✧ The recovered Si meets the specifications for solar-grade Si; the recovery rate is 90%.	2017/[76]
	60–80 °C, KOH (30%), 2–3 min, 40 °C, HNO_3_ (65%), HF (40%), CH_3_COOH (99.5%) + Br_2_, 9 s.	✧ Etching solutions need to be modified according to the type of PV cells to be recycled.	2010/[12]
	RT, HF (48%), HNO_3_ (70%), H_2_SO_4_ (97%), CH_3_COOH (99%), Surfactant of CMP-MO-2 (20 wt%), 20 min.	✧ The recovery purity of silicon is 99.999% with a recovery rate of 86%, ✧ The addition of surfactant improves the recovery rate.	2012/[115]
	160 °C, H_3_PO_4_ (90%), 60 min, RT, HNO_3_ (60%): HF (49%) = 4:1 (*v*/*v*), 1 min.	✧ The recycled silicon wafers demonstrate comparable properties to commercial virgin wafers.	2014/[116]
	RT, HCl (50%), 30 min, RT, HF (10%), 10 min, Al-Si solvent refining: Al (99.996%), 1000 °C, 10 h, HCl.	✧ The recovered Si meets the specifications for solar-grade Si.	2025/[117]
	RT, HCl (18–24 wt%), 10–15 min, 50 °C, HNO_3_ (30 wt%), 10 min, RT, HF (20–30 wt%), 5 min, RT, Cu (NO_3_)_2_, AgNO_3_, HF, H_2_O_2_, 5 min, RT, HNO_3_ (30 wt%), Ultrasonic, 15 min, 80 °C, HCl/H_2_O_2_/H_2_O (1/1/6), 20 min, 50 °C, KOH (0.2 mol/L), (CH_3_)_2_CHOH (10 vol%), 90 s.	✧ Recycled silicon wafers offer superior performance to commercial products alongside lower production costs than conventional industrial wafers.	2022/[57]
	RT, HNO_3_ (5 mol/L), 1 h, 160 °C, H_3_PO_4_ (90%), 1 h, 80 °C, KOH (45%), 10 min.	✧ Recovery rate of Si is 80% and free HF.	2016/[30]
	RT, HNO_3_ (60%), 5 min, 80 °C, KOH (45%), 8 min, 320 °C, etching paste containing H_3_PO_4_, 2 min, KOH (0.05%).	✧ The recycled silicon wafers demonstrate comparable properties to commercial virgin wafers and free HF.	2017/[118]
	RT, HNO_3_ (60%), 10 min, 180 °C, H_3_PO_4_ (90%), 30 min, 80 °C, NaOH (45%), 5 min.	✧ Recovered Si meets the requirements for silicon anodes in LIBs and free HF, ✧ Providing 2427.7 mAh/g capacity after 200 cycles.	2021/[119]
	63 °C, NaOH (10 mol/L), 5 min, 70 °C, HNO_3_ (6 mol/L), 5 min, 150 °C, H_3_PO_4_ (90%), 45 min.	✧ The recovery purity of silicon is 99.9984% and free of HF.	2021/[120]
	RT, HNO_3_ (60%), 2 min, Mechanical grinding, 20 RPM, 20 min, 80 °C, KOH (45%), 10 min.	✧ Use mechanical grinding to replace HF etching, ✧ The recycled silicon wafers demonstrate comparable properties to commercial virgin wafers.	2016/[121]
	Molten-salt-etching, 200 °C, NaOH (42 wt%) + KOH (58 wt%), 2 s, 200 °C, salt etchin, 1–3 min, Water washing, 70 °C, NaOH, 0.5–5 min.	✧ Using molten salt etching to replace strong acid etching enables rapid purification and recovery of silicon, ✧ Recovery rate of Si is 98%.	2024/[122]

**Table 8:** Recovery technologies for valuable metals from c-Si PV cells.

Technology	Process and Key Parameters	Target Metal	Key Points	Year/References
Acid leaching	RT, HNO_3_ (60%), 2 h, NaCl (99%).	Ag	✧ Silver recovery is 94%.	2016/[124]
	RT, HNO_3_ (5 mol/L), 1 h, Cu^2+^: Extraction, LIX84-I (20%), H_2_SO_4_ (150 g/L), Electrowinning, 0.5 A/dm^2^, 24 h, Ag^+^: HCl (5 mol/L), NaOH (5 mol/L), N_2_H_4_·H_2_O, Electrowinning, 0.5 A/dm^2^, 24 h.	Ag, Cu	✧ Silver recovery is 90% (purity > 99%), ✧ Copper recovery is 79%, ✧ Removing Pb through neutralization and sulfidation processes (93% removal).	2016/[30]
	HNO_3_ (30%), Electrowinning, Ag^+^: 0.3 V (vs. Ag/AgCl), 5.5 h, Cu^2+^: −0.3 V (vs. Ag/AgCl), 24 h.	Ag, Cu	✧ Silver recovery is 74% (purity > 99%), ✧ Copper recovery is 83% (purity > 99%).	2017/[76]
	80 °C, HNO_3_ (5 mol/L), 1 h, Ag^+^: NaCl, NaOH, Glucose, Sn^2+^: Extraction, TBP (30%), HNO_3_ (1 mol/L), NH_4_OH, Calcination, Cu^2+^: Extraction, LIX984N (5%), H_2_SO_4_ (3 mol/L), NH_4_OH, Calcination.	Ag, Cu, Sn	✧ Silver recovery is 99.7% (98.85% purity), ✧ Copper recovery is 98.5% (including Cu and CuO), ✧ Tin recovery is 96.5% (SnO_2_), ✧ Copper strips are recovered from photovoltaic solders, ✧ Pb is Removed.	2021/[114]
	RT, MSA (99 wt%): H_2_O_2_ (30 wt%) = 90: 10 (*v*/*v*), 4 h, Ag^+^: HCl (35 wt%), NaOH (2 wt%), H_2_O_2_ (30 wt%), Electrowinning.	Ag	✧ The recovery purity of silver is 99.995%, ✧ MSA is environmentally friendly and reusable.	2017/[125]
	Electrochemical-assisted, H_2_SO_4_ (5 mol/L), 100–300 mA/cm^2^, Electrowinning, BDD electrode, Ag^+^: 0.8 V (vs. SHE); Cu^2+^: 0.35 V (vs. SHE).	Ag, Cu	✧ Silver recovery is 88%, ✧ Copper recovery is 99%.	2021/[126]
DES leaching	75 °C, Ethaline DES (ChCl:EG = 1:2) + FeCl_3_·6H_2_O (0.12 mol/L), Electrowinning, 50 °C, −0.47 V (vs. Fe^2+^/Fe^3+^), Argon atmosphere.	Ag	✧ 99.9% silver leaching efficiency, ✧ The recovery purity of silver is 98.8%, ✧ An oxygen-free environment is crucial for silver electrodeposition.	2024/[127]
Salt-etching	200 °C, Molten NaOH-KOH (0.42:0.58), 1–3 min, Water washing, Solder, Surface oxidation, 500 °C, 1 h, 90 °C, NaOH (50 wt%), Electrowinning.	Ag, Cu, Sn, Pb	✧ Silver recovery is 99.0% (99.5% purity), ✧ Silver can be recovered quickly.	2024/[122]
Others	Leaching, I_2_ (0.35 mol/L), KI (0.7 mol/L), 5 min, Al^3+^: Acetate and borate, pH = 9.6, Ag^+^: Zinc cementation.	Ag, Al	✧ More than 95% of silver and aluminum can be leached within the first 5 min, ✧ Low environmental impact.	2021/[128]
	69 °C, NaOH (2 mol/L), 6 min, 70 °C, FeCl_3_ (0.8 mol/L), 20 min.	Ag	✧ Silver recovery is 93.0%, ✧ Low environmental impact.	2025/[129]

## Data Availability

The original contributions presented in the study are included in the article; further inquiries can be directed to the corresponding author.

## Abbreviations

The following abbreviations are used in this manuscript:

Al-BSF	Aluminum back surface field
AR	Anti-reflective
CPCB	Central Pollution Control Board (India)
c-Si	Crystalline Silicon
DBE	Dibasic ester
DEM	Diethyl malonate
DES	Deep eutectic solvents
DMC	Dimethyl carbonate
DMAC	N,N-Dimethylacetamide
DMPU	N,N′-Dimethylpropenylurea
EG	Ethylene glycol
EGDA	Ethylene glycol diacetate
EHD	Electro-hydraulic disintegration
EHF	Electro-hydraulic fragmentation
EL	Electroluminescence
ElektroG	Electrical and Electronic Equipment Act (Germany)
EMAA	Ethylene-methacrylic acid
EOL	End-of-life
EPR	Extended producer responsibility
EVA	Ethylene-vinyl acetate
FIT	Feed-in tariff
IBC	Interdigitated back contact
IEA	International Energy Agency
IEA-PVPS	International Energy Agency Photovoltaic Power Systems Programme
IEC	International Electrotechnical Commission
IPM	Isopropyl myristate
IRENA	International Renewable Energy Agency
LiBs	Lithium-ion batteries
LIX84-I	2-hydroxy-5-nonylacetophenone oxime
LIX984N	A 1:1 volume blend of 5-nonylsalicylaldoxime and 2-hydroxy-5-nonylacetophenone oxime
MACE	Metal-assisted chemical etching
Mc-Si	Multicrystalline silicon
METI	Ministry of Economy, Trade and Industry (Japan)
MNRE	Ministry of New and Renewable Energy (India)
MOE	Ministry of Environment (Japan)
Mono-Si	Monocrystalline Silicon
MSA	Methanesulfonic acid
MST	Module safety test
NDRC	National Development and Reform Commission
NMP	N-methyl-2-pyrrolidone
NPV	Net Present Value
PAS	Publicly Available Specification
PERC	Passivated emitter and rear cell
PID	Potential induced degradation
PLR	Performance loss rate
POE	Polyolefin
POLO	Polysilicon on oxide
Poly-Si	Polysilicon
PROs	Producer Responsibility Organizations
PV	Photovoltaic
PVDF	Polyvinylidene Fluoride
PVF	Polyvinyl fluoride
RCRA	Resource Conservation and Recovery Act (US)
RT	Room temperature
SC-CO_2_	Supercritical carbon dioxide
SHE	Standard hydrogen electrode
SHJ	Silicon heterojunction
TOPCon	Tunnel oxide passivating contact
TPA	Terephthalic acid
TPT	Tedlar Polyester Tedlar
WEEE	Waste Electrical and Electronic Equipment Directive
